# Genetic diversity and differentiation among insular honey bee populations in the southwest Indian Ocean likely reflect old geographical isolation and modern introductions

**DOI:** 10.1371/journal.pone.0189234

**Published:** 2017-12-27

**Authors:** Maéva Angélique Techer, Johanna Clémencet, Christophe Simiand, Patrick Turpin, Lionel Garnery, Bernard Reynaud, Hélène Delatte

**Affiliations:** 1 Université de La Réunion, UMR PVBMT, La Réunion, France; 2 CIRAD, UMR PVBMT, Saint Pierre, La Réunion, France; 3 Université de Versailles Saint-Quentin-en-Yvelines, Versailles, France; National Cheng Kung University, TAIWAN

## Abstract

With globalization the Western honey bee has become a nearly cosmopolitan species, but it was originally restricted to the Old World. This renowned model of biodiversity has diverged into five evolutionary lineages and several geographic “subspecies.” If *Apis mellifera unicolor* is indubitably an African subspecies endemic to Madagascar, its relationship with honey bees from three archipelagos in the southwest Indian Ocean (SWIO) hotspot of biodiversity is misunderstood. We compared recent mtDNA diversity data to an original characterization of the nuclear diversity from honey bees in the Mascarenes and Comoros archipelagos, using 14 microsatellites, but also additional mtDNA tRNA^Leu^-cox2 analysis. Our sampling offers the most comprehensive dataset for the SWIO populations with a total of 3,270 colonies from 10 islands compared with 855 samples from Madagascar, 113 from Africa, and 138 from Europe. Comprehensive mitochondrial screening confirmed that honey bees from La Réunion, Mauritius, and Comoros archipelagos are mainly of African origin (88.1% out of 2,746 colonies) and that coexistence with European lineages occurs only in the Mascarenes. PCA, Bayesian, and genetic differentiation analysis showed that African colonies are not significantly distinct on each island, but have diversified among islands and archipelagos. *F*_ST_ levels progressively decreased in significance from European and African continental populations, to SWIO insular and continental populations, and finally among islands from the same archipelago. Among African populations, Madagascar shared a nuclear background with and was most closely related to SWIO island populations (except Rodrigues). Only Mauritius Island presented clear cytoplasmic disequilibrium and genetic structure characteristic of an admixed population undergoing hybridization, in this case, between *A*. *m*. *unicolor* and *A*. *m*. *ligustica*, *A*. *m*. *carnica* and *A*. *m*. *mellifera*-like individuals. Finally, global genetic clustering analysis helped to better depict the colonization and introduction pattern of honey bee populations in these archipelagos.

## Introduction

Islands are rich reservoirs of biodiversity with high endemism across diverse taxonomic groups [1–3]. Often referred as nature’s test tubes [4], these isolated environments are less complex than continents, and unique [5–7]. Island populations are often characterized by relatively low genetic diversity, possibly resulting from i) founder effect and bottleneck, ii) small effective population sizes, iii) geographic isolation [8], and/or progressive archipelago colonization [9]. Among the 35 revised worldwide vulnerable hotspots of biodiversity [10], Madagascar and the southwest Indian Ocean (SWIO) islands shelter high rates of endemism [2, 10]. The islands surrounding Madagascar are part of the Mascarenes Archipelago (La Réunion, Mauritius, and Rodrigues) in the East, the Seychelles Archipelago (Mahé, Praslin, La Digue main islands) in the Northeast and the Comoros Archipelago (Grande Comore, Mohéli, Anjouan, and Mayotte) in the Northwest.

In these rich endemic ecosystems, species such as the Western honey bee, *Apis mellifera*, a generalist pollinator, deserve particular attention. The honey bee is established in all three SWIO archipelagos and, has the particularity to occur both in wild and domesticated states. If multiple livestock species are known to be exotic and deliberately brought to all these islands by human [11], the case of *A*. *mellifera* is not as obvious. Genomic analysis suggested that *A*. *mellifera* originated and colonized its native geographic range—Africa, Europe, the Middle East, and some regions in Asia—at least 300,000 years ago [12]. Following multiples colonization waves and glaciation events, *A*. *mellifera* diverged into five evolutionary lineages [13–18]. Apart from the European M and C, Oriental O, and Yemenite Y lineages, the largest African lineage subdivided into A_I_, A_II_, A_III_ and Z sub-lineages [19] with a split estimated at 32,700 to 23,000 years ago between African subspecies [12]. Prehistoric pottery analysis revealed that Human started to interact with *A*. *mellifera* for beeswax for almost 9,000 years [20, 21]. Semi-domestication of honey bee has surely influence its genetic diversity via global movements and artificial selection [22, 23]. Among the 31 subspecies commonly used in the *Apis* literature [24–31], *A*. *m*. *unicolor* has been described as endemic to Madagascar [13]. Intra-species divergence and human colonization dating (first evidence of human settlement was dated ~4380–4940 years ago [32]) suggest that *A*. *m*. *unicolor* colonized Madagascar well before human arrival. Nevertheless, the hypothesis of natural colonization by *A*. *mellifera* into the nearby SWIO archipelagos is still questioned, especially regarding to botanical studies that consider it to be introduced from Madagascar into the nearby SWIO archipelagos (based on divergent historical records [33–36]).

Recent combined analysis of both the ND2 gene and the tRNA^Leu^-cox2 intergenic region from mtDNA supported an insular African sub-group in the SWIO islands, distinct from continental sub-lineages [37]. The presence of *A*. *m*. *unicolor* in the Mascarenes (except Rodrigues), Comoros, and Seychelles archipelagos was shown by shared haplotypes with Madagascar [37]. Hints of ancient colonization and diversification within the SWIO region was supported by private tRNA^Leu^-cox2 diversity on each island. Despite mtDNA similarities, using microsatellite markers, honey bee populations between the Seychelles Archipelago and Madagascar (separated by 1,100 km of ocean) were found to be genetically differentiated [38]. In the Mascarenes, proportions of mtDNA haplotypes from the European lineage drastically varied from an island to another [37] reaching an exclusive level in the eastern Rodrigues population [39]. These findings confirmed that multiples introductions occurred in the Mascarenes but their effect on La Réunion and Mauritius populations have never been characterized using nuclear markers. Therefore, it is unknown whether these populations are undergoing hybridization. Mitochondrial DNA sequencing indicated that Comoros Archipelago might act as a contact area between the Africa coast and Madagascar, and require to be carefully examined using nuclear markers.

The present study characterizes for the first time, the nuclear genetic diversity of honey bee populations within La Réunion and Mauritius (Mascarenes Archipelago) and populations found in Grande Comore, Mohéli, Anjouan, and Mayotte Islands (Comoros Archipelago). We compared the mtDNA polymorphism with nuclear diversity and structuration help in the detection of ongoing hybridization between subspecies on La Réunion and Mauritius. Since multiples lineages coexist in these islands, processes shaping the genetic diversity are hard to disentangle without a large and diverse dataset, capable of discerning between African and European populations. For that reason, we implemented additional sampling from continental native populations and previous datasets from Madagascar [40], Seychelles [38], and Rodrigues [39], to assess the relationship among insular and continental populations. Using the most comprehensive genetic dataset of *A*. *mellifera* in the SWIO, we propose an interpretation of the intraspecific phylogeographic patterns in the three archipelagos.

## Material and methods

### Honey bee population sampling

**Fig 1:**
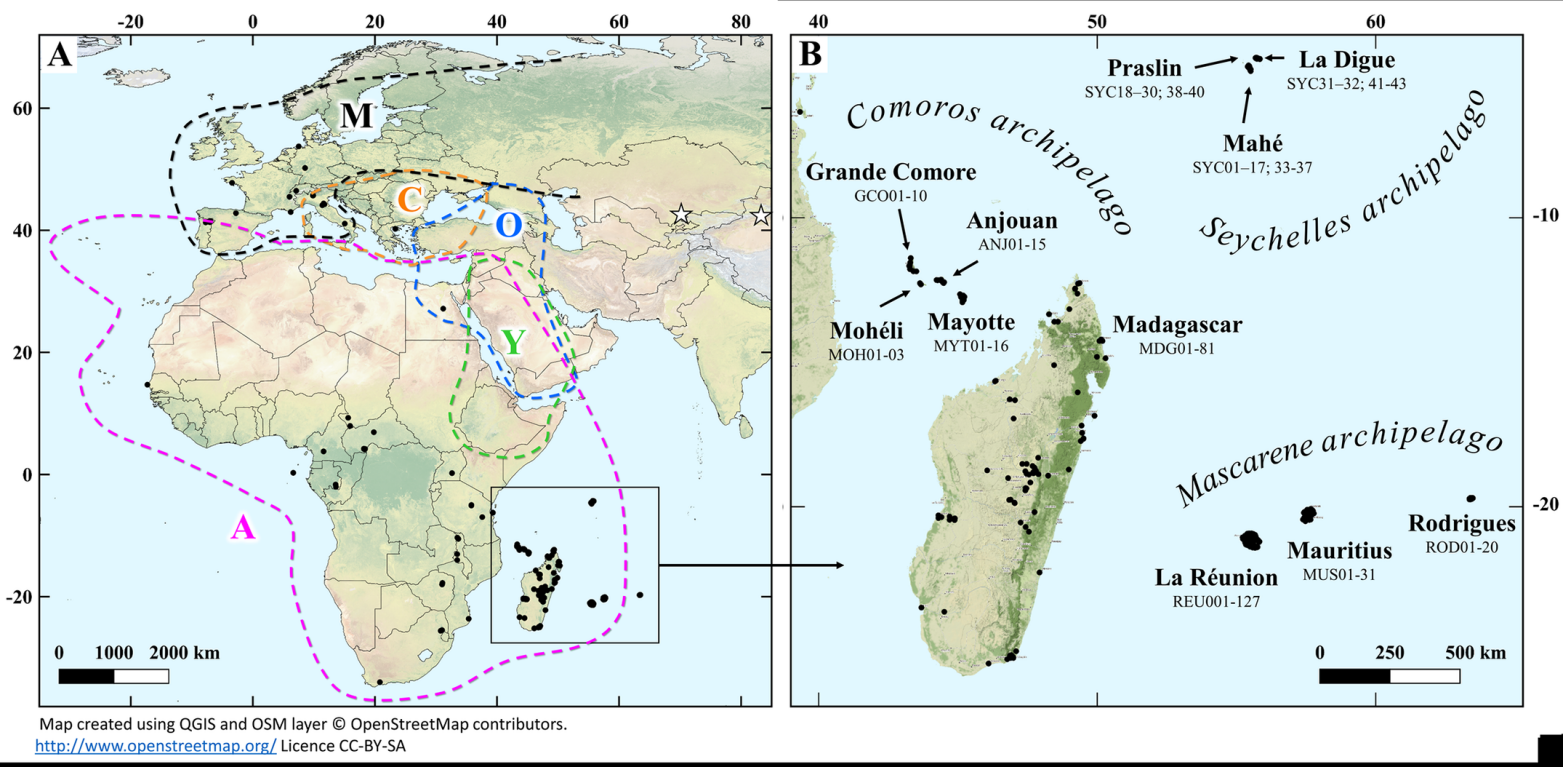
A) Geographical distribution of honey bee sampling sites in Africa, Europe, and islands of the southwest Indian Ocean (SWIO) and B) Geographic location of the Mascarene, Seychelles, and Comoros Archipelagos with respect to Madagascar. Sample locations are represented by circles. Approximate distributions of five evolutionary lineages of the Western honey bee (A, C, M, O and Y) are delimitated by dashed lines. White stars showed the location of the two far-eastern subspecies, *A. m. pomonella* [24] and *A. m. sinisxinyuan* [27]. Map layer from the open source, OpenStreetMap.

Genetic diversity in *A*. *mellifera* populations from the SWIO islands was assessed by *de novo* genotyping of worker honey bees from 2,860 colonies from both insular and continental areas. In order to have a comprehensive understanding of SWIO honey bee phylogeography, a dataset containing 1,528 individuals formerly described in Madagascar, Seychelles, Rodrigues, South Africa, and Italy was also employed [37] (Table 1). The sample (*N* = 4,388) covered honey bee populations in the SWIO islands and different habitats throughout Africa and Europe (Fig 1, S1 and S2 Figs).

**Table 1:** Location details for populations sampled in islands of the southwest Indian Ocean, Africa, and Europe. *N de novo* the number of honey bee colonies newly sampled, dataset μsat & tRNA^Leu^-cox2: number of colonies for which microsatellites and mtDNA tRNA^Leu^-cox2 dataset were previously described, with associated references in brackets, dataset tRNA^Leu^-cox2: number of colonies for which only mtDNA (tRNA^Leu^-cox2 dataset) was previously described, but microsatellites were analyzed only in this study.

	Site	# of sites	Sampling date	*N de novo*	Dataset μsat & tRNA^Leu^-cox2	Dataset tRNA^Leu^-cox2	Total
**Southwest Indian Ocean islands**							
Madagascar	MDG01-76	76	2011–2013		748 [40]		**748**
	MDG77	1	2014	7		5 [37]	**12**
	MDG78-81	4	1996–1998	78		17 [37]	**95**
***Mascarenes Archipelago***							
La Réunion	REU001-127	127	2011–2012	1920		130 [37]	**2050**
Mauritius	MUS01-31	31	2012–2014	128		239 [37]	**367**
Rodrigues	ROD01-20	20	2013		524 [39]		**524**
***Seychelles Archipelago***							
Mahé	SYC01-17	17	2013		71 [38]		**71**
	SYC33-37	5	2015	10			**10**
Praslin	SYC18-30	13	2013		71 [38]		**71**
	SYC38-40	3	2015	6		2 [37]	**8**
La Digue	SYC31-32	2	2013		43 [38]		**43**
	SYC41-44	4	2015	6			**6**
***Comoros Archipelago***							
Grande Comore	GCO01-10	10	2013			29 [37]	**29**
Mohéli	MOH01-03	3	2013	1		10 [37]	**11**
Anjouan	ANJ01-15	15	2013–2015	18		27 [37]	**45**
Mayotte	MYT01-16	16	2012	11		24 [37]	**35**
**African populations**							
Egypt	EGY01	1	1997	1		1 [37]	**2**
Senegal	SEN01	1	2015			2 [37]	**2**
São Tomé Island	STP01	1	1998	3		9 [37]	**12**
Chad	TCD01-02	2	2015	1		3 [37]	**4**
Central African Republic	CAF01-05	5	2013–2015			11 [37]	**11**
Cameroon	CMR01	1	2015	6			**6**
Gabon	GAB01-02	2	2014			3 [37]	**3**
Uganda	UGA01	1	2015			1 [37]	**1**
Malawi	MWI01-04	4	1995	4		4 [37]	**8**
Tanzania	TZA01-03	3	2015	4		10 [37]	**14**
Tanzania Zanzibar	ZAN01	1	2015	3			**3**
Zimbabwe	ZWE01	1	1995			5 [37]	**5**
	ZWE02	1	2014			9 [37]	**9**
Mozambique	MOZ01	1	2015			2 [37]	**2**
South Africa	ZAF01-03	3	2013–2015	9	22 [40]		**31**
**European populations**							
Switzerland	CHE01	1	2013	1		2 [37]	**3**
Germany	DEU01	1	1998	4		2 [37]	**6**
	DEU02	1	2013	2		1 [37]	**3**
Italy	ITA01-08	8	1997	7	49 [38]		**56**
Greece	GRC01	1	2015			6 [37]	**6**
France	FRA01-03	3	2013	20		28 [37]	**48**
Spain	ESP01	1	2013			3 [37]	**3**
Portugal	PRT01-06	6	2013	1		12 [37]	**13**
**Pacific insular population**							
Tahiti	TAH01	1	2013	12			**12**
		**398**		**2,263**	**1,528**	**597**	**4,388**

Sampling efforts focused on previously undescribed populations using nuclear markers from La Réunion, Mauritius, Grande Comore, Mohéli, Anjouan, and Mayotte Islands. To increase the probability of obtaining samples representative of these populations, to the extent possible, colonies were collected in different habitats across each island. For La Réunion and Mauritius, collection sites encompassed urban areas as well as virgin tropical forest in National Parks (Parc National de La Réunion and Black River Gorges National Park, respectively). 15.8% of the known managed honey bee populations in La Réunion (13,000 managed colonies; GDS, 2014 *personal communication*) and 13.6% of those in Mauritius (2,700 colonies; Jhumun [41]) were sampled. Beekeeping is poorly developed in the Comoros Archipelago, so that honey bee foragers were collected every 5 km, whenever it was possible. For each managed or wild colony, workers were collected at the entrance or inside each colony. Immersion in 95% ethanol immediately killed workers, and they were stored at -20°C until laboratory processing.

Sampling of honey bee colonies in continental Africa and Europe provided reference native populations for comparison. A total of 113 colonies were sampled at 28 locations in the known ranges of continental African subspecies, *A*. *m*. *adansoni*, *A*. *m*. *lamarckii*, *A*. *m*. *scutellata*, *A*. *m*. *capensis*, *A*. *m*. *monticola*, and *A*. *m*. *litorea*. In Europe, 138 colonies were sampled at 22 locations covering known habitats of the two M lineage subspecies (*A*. *m*. *iberiensis* and *A*. *m*. *mellifera*) and three C lineage subspecies (*A*. *m*. *carnica*, *A*. *m*. *ligustica*, *A*. *m*. *cecropia*). Finally, 12 colonies to a strictly known introduced insular population from Tahiti were collected.

All maps depicting sampling lovations were generated and using QGIS software [42] and derived from open source layers OSM and the world border country polygon shapefile freely available from https://github.com/petewarden/openheatmap/tree/master/mapfileprocess/test_data/TM_WORLD_BORDERS-0.3.

### Maternal lineage identification using mtDNA tRNA^Leu^-cox2 PCR-RFLP

Total DNA was extracted from legs of one honey bee per colony as described in [37]. Ancestral evolutionary lineage was determined using the rapid and standardized PCR-RFLP on the tRNA^Leu^-cox2 intergenic region [43]. Amplification employed E2 and H2 primers [14]. Then enzymatic digestion using *DraI* was performed following manufacturer recommendations (*Promega*). tRNA^Leu^-cox2 amplified, and restriction fragments were visualized on 4% agarose gels and recorded. All restriction profiles detected were already described in *A*. *mellifera* populations, easing their identification.

### Genotyping of workers using microsatellites

All DNA samples were amplified using multiplex PCR reactions with 14 microsatellite loci: A113, A24, AP55, A88, A28, A29, AP289, AP273, (A)B124, A8, A35, AP33, AP66, and AP43 [44–47]. Multiplex composition, PCR reactions, and genotype scoring employed the same conditions used for the comparative microsatellite dataset from the Rodrigues [39], Madagascar [40] and Seychelles populations [38]. An individual was considered adequately genotyped when ≥ 60% of multilocus genotyping was successful. Potential genotyping errors were checked using MICRO-CHECKER 2.2.3 [48].

### Population genetic analysis

Genetic diversity was analyzed at both fine and coarse scales by considering different population levels with at least five individuals per apiary. Intra-island/country genetic variation was estimated using each site as a population unit (398 sites) while inter-insular/continental analysis considered all sites from the same island/country (11 islands and 21 countries). Null allele frequency per locus (A_null_) was estimated for each population unit with FREENA [49]. The mean number of alleles (N_all_), observed heterozygosity (H_obs_), unbiased expected heterozygosity (H_nb_), and *F*_IS_ per population unit were estimated using GENETIX 4.05 [50]. Distributions of alleles within and among insular and continental populations was calculated using ADZE [51]. Allelic richness was computed and tested with R and the PopGenReport package [52] using the rarefaction method (for each island/country with a minimum of 6 diploid individuals). Population unit pairwise *F*_ST_s were estimated using FSTAT 2.9.3.2 software [53]. GENEPOP 3.4 was used to test deviation from Hardy-Weinberg (HW) equilibrium and genetic differentiation between population pairs [54]. Regarding comparisons among pairs of insular and continental populations, only French sites were kept as separated populations due i) to divergence of mtDNA and ii) the nuclear background shown by significant *F*_ST_ values.

Principal Component Analysis (PCA) was used to further explore genetic differentiation between populations, using R 3.0.2 software [55] and adegenet 1.3–9.2 package [56]. Genetic structure among populations was additionally investigated using STRUCTURE 2.3.3 [57]. A total of 10^6^ simulations using 10^5^ burn-in steps and MCMC (Markov Chain Monte Carlo algorithm) steps were run for all samples (*N* = 4,388) simultaneously, considering a K interval [1–30] with ten iterations each. The optimal number of clusters was estimated using the ΔK method of Evanno [58]. In parallel, INSTRUCT software [59], which takes into account inbreeding, was run with the same parameters to confirm results from STRUCTURE. Discriminant Principal Components Analysis (DAPC) was also used to support population structure results [60]. Subsequent runs were performed to evaluate intra-island structure in i) La Réunion sites (*N* = 2,050), ii) Mauritius (*N* = 367), iii) islands of the Comoros Archipelago (*N* = 120) and iv) continental populations (*N* = 263). STRUCTURE HARVESTER [61], CLUMPP [62] and DISTRUCT 1.1 [63] were used to develop the graphical output.

## Results

Full sample details including sampling geo-coordinates, multilocus genotypes using the mtDNA tRNA^Leu^-cox2 intergenic region, and 14 *loci* microsatellites are available in S1 Table.

### Distribution of mitochondrial evolutionary lineages

Successful analysis of the tRNA^Leu^-cox2 intergenic region identified 19 restriction profiles in 4,252 colonies from SWIO, Africa, Europe, and Tahiti honey bee populations. The African lineage was characterized by A1, A4, A6, A11, A14, A16, A48, A49, A50, A51, Z2 and Z7 profiles while the European M lineage was distinguished by M3, M4, M6, M7, M7’ and M65. On gels, C1 and C2 profiles were difficult to discriminate (1bp difference), so those were coded as C1/C2, but both indicated the European C lineage.

In the SWIO, the distributions and proportions of honey bee mtDNA evolutionary lineages were similar to those reported previously based upon smaller sampling [37]. In Madagascar, Mahé, Praslin, La Digue, Grande Comore, Mohéli, Anjouan and Mayotte, all colonies had tRNA^Leu^-cox2 *DraI* profiles characteristic of the African lineage. The Mascarenes Archipelago was the only region in the SWIO to exhibit three different lineages: i) in La Réunion, 95.5% lineage A, 4.3% C, and 0.3% M; ii) in Mauritius, 54.2% A, 44.7% C and 1.1% M; iii) in Rodrigues, 100% C. Proportions of evolutionary lineages formerly reported for the Mascarenes using tRNA^Leu^-cox2 sequencing (*N* = 130 for La Réunion and *N* = 239 for Mauritius [37]) was largely confirmed, despite a massive difference in sample size (*N* = 2,050 for La Réunion and *N* = 367 for Mauritius). As for continental populations, distribution of tRNA^Leu^-cox2 restriction profiles shifted from A maternal lineages in African colonies to M and C maternal lineages in European colonies (Table 2).

**Table 2:** Evolutionary lineage occurrence and microsatellite genetic diversity indices in each insular and continental honey bee population. For each *DraI* restriction profile of the mtDNA tRNA^Leu^-cox2 amplified fragment (P_0_Q, P_0_QQ, P_1_QQ, P_1_QQQ, PQ, PQQ and Q length), corresponding evolutionary lineages (A, M, C) are detailed. Nμsat: number of successfully genotyped workers; NA _tRNALeu-cox2:_ number of individuals with tRNA^Leu^-cox2region missing data, N_all_: mean number of alleles; H_obs_: observed heterozygosity; and A_null_: mean null allele frequency.

	A												M						C					
	P_0_Q				P_0_QQ					P_1_QQ		P_1_QQQ	PQ		PQQ			PQQQ	Q					
	A1	A48	A49	Z7	A4	A6	A50	A51	Z2	A11	A14	A16	M3	M6	M4	M65	M7	M7’	C1/C2	NA _tRNALeu-cox2_	Nμsat	*N*_*all*_	*H*_*obs*_	*A*_*null*_
***SWIO islands*:**																								
Madagascar	850	2			3																**855**	9.57	0.414	0.022
La Réunion	1886				5										5				85	69	**2,050**	16.21	0.667	0.011
Mauritius	196		2		1												4		164		**367**	10.57	0.550	0.022
Rodrigues																			468	56	**524**	7.64	0.648	0.001
Mahé	81																				**81**	7.00	0.572	0.015
Praslin	75								4												**79**	5.57	0.554	0.014
La Digue	45								4												**49**	5.00	0.510	0.014
Grande Comore	**29**																				**29**	4.57	0.480	0.024
Mohéli	**11**																				**11**	3.85	0.447	0.083
Anjouan	**45**																				**45**	4.42	0.492	0.024
Mayotte	**35**																				**35**	3.57	0.452	0.031
***Africa*:**																								
Egypt				2																	**2**	-	-	-
Senegal					2																**2**	-	-	-
São Tomé Island					10		1													1	**12**	4.79	0.693	0.014
Chad					2	1														1	**4**	-	-	-
CAF	6				5																**11**	9.64	0.864	0.021
Cameroon	3				2															1	**6**	6.42	0.767	0.028
Gabon	2				1																**3**	-	-	-
Uganda								1													**1**	-	-	-
Malawi	5																			3	**8**	7.64	0.830	0.024
Mozambique	2																				**2**	-	-	-
Tanzania	10				4																**14**	9.50	0.775	0.034
Zanzibar	3																				**3**	-	-	-
Zimbabwe					14																**14**	10.29	0.82	0.03
South Africa	2				25		1													3	**31**	13.14	0.823	0.023
***Europe*:**																								
Switzerland																			2	1	**3**	-	-	-
Germany																			9		**9**	4.14	0.516	0.031
Italy													6				19	1	30		**56**	7.21	0.475	0.001
Greece																			6		**6**	3.21	0.452	0.027
France														6	20	3		4	15		**48**	9.36	0.601	0.054
Spain										2								1			**3**	-	-	-
Portugal	2									7	1	3									**13**	5.07	0.517	0.003
***Pacific island*:**																								
Tahiti																	7		4	1	**12**	3.85	0.330	0.023
**Total**	**3,288**	**2**	**2**	**2**	**74**	**1**	**2**	**1**	**8**	**9**	**1**	**3**	**6**	**6**	**25**	**3**	**30**	**6**	**783**	**136**	**4,388**			

### Nuclear genetic diversity in insular and continental honey bee populations

**Fig 2:**
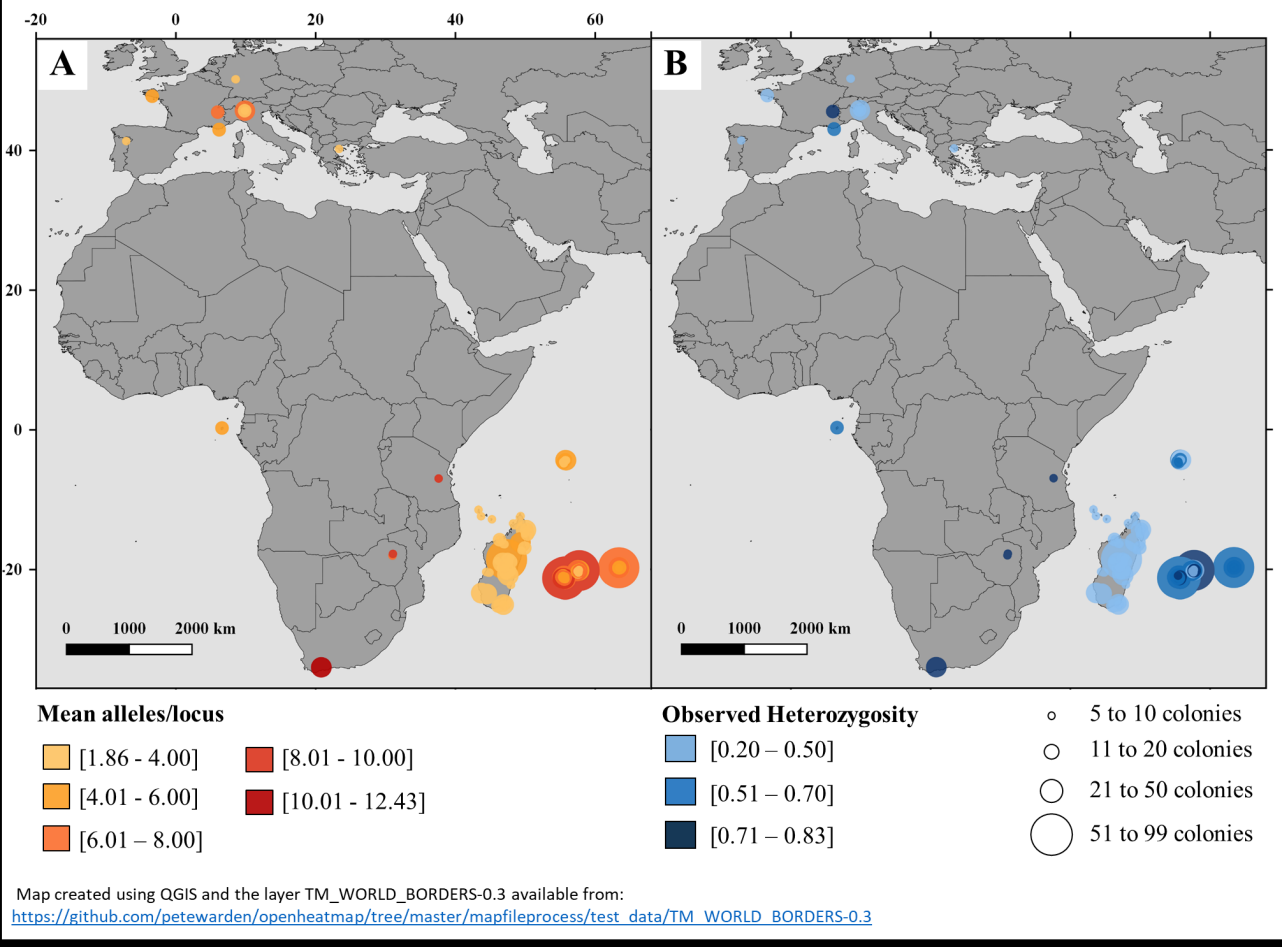
Distribution of genetic diversity of honey bee populations (*n* ≥ 5) from the southwest Indian Ocean islands compared to the native continental range. (A) Mean number of alleles per locus (14 microsatellites). (B) Gradients in observed heterozygosity levels per site and sample sizes.

**Fig 3:**
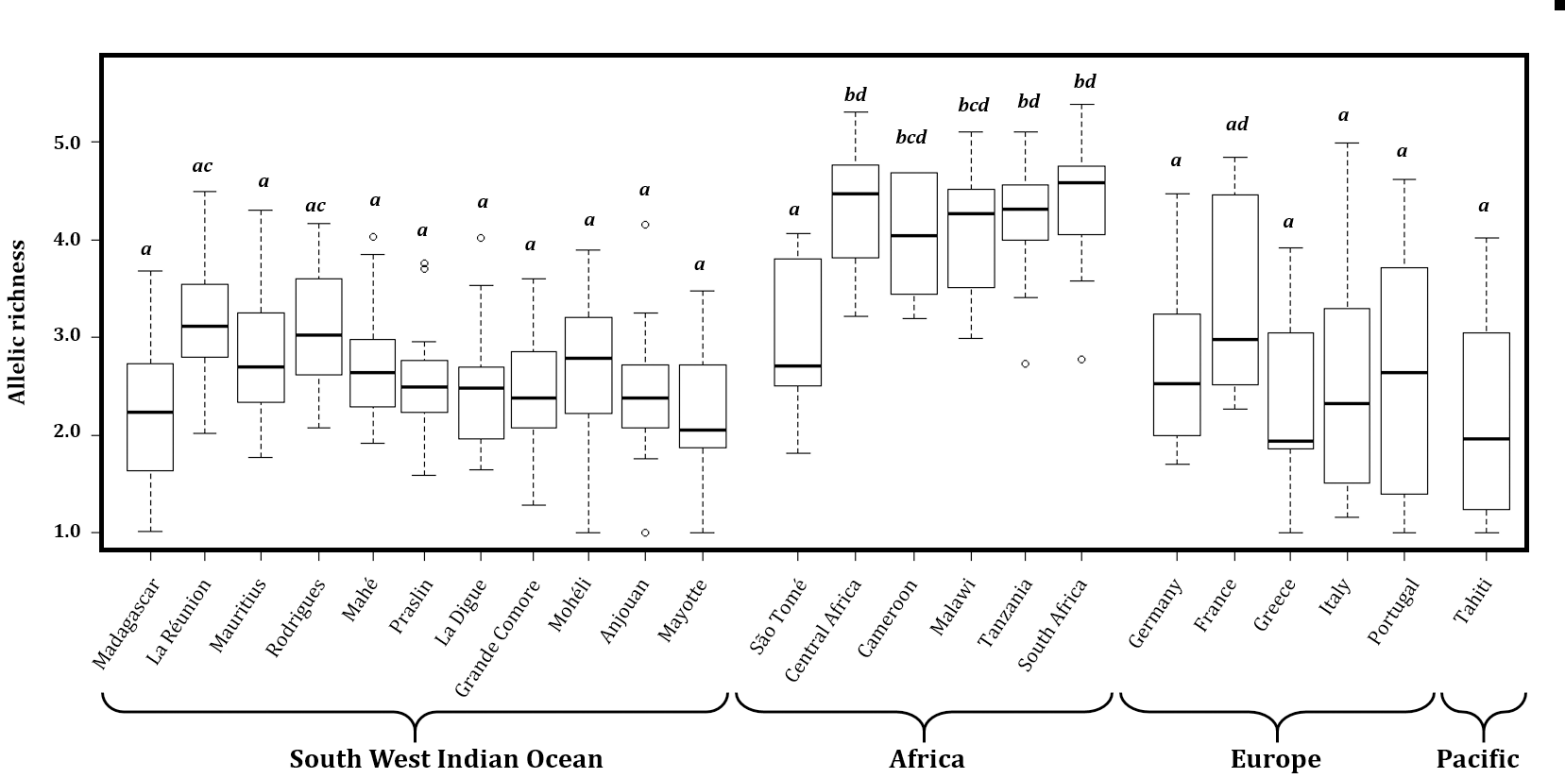
Allelic richness observed in southwest Indian Ocean honey bees is comparable to that of native continental European populations, but still less than that of African populations. Boxplot of allelic richness using rarefaction for insular and continental honey bee populations (minimum of 6 diploid individuals) in the SWIO, Africa, Europe, and Tahiti. Boxes with the same letter do not differ significantly (P > 0.05 ANOVA followed by Tukey’s HSD test).

All samples (*N* = 4,388) were considered successfully genotyped (with less than 40% missing data). Preliminary analysis showed negligible low null allele frequencies for all insular and continental populations listed in Table 2 (site details are given in S2 Table). The asymptotic trend observed in allele accumulation curves may indicate that the majority of alleles at the 14 studied microsatellites *loci* were captured in the La Réunion and Mauritius populations (S3 Fig). The mean number of alleles per site (*n* ≥ 5) showed that African and Mascarenes honey bees have the highest genetic diversity of the populations tested (Fig 2A). However, when looking more closely at La Réunion and Mauritius populations, the mean number of alleles seems to be related to site sampling size (S4 Fig). Allelic richness using rarefaction was more appropriate to confirm this pattern due to the sample size difference. The SWIO insular population allelic richness was not significantly different from continental European populations, but was significantly lower than continental African ones. This lower allelic richness was likewise observed for another insular population in São Tomé (Ar = 2.94 ± 0.71). In the Mascarenes Archipelago, only La Réunion (Ar = 3.18 ± 0.71) and Rodrigues (Ar = 3.09 ± 0.67) displayed allelic richness levels comparable to those of the Cameroon and Malawi populations (Fig 3).

Heterozygosity levels in the SWIO islands were heterogeneous regardless of sample size (Fig 2B, S2 Table). Compared to Madagascar (41.4% heterozygosity), La Réunion (66.7%) and Rodrigues (64.8%) possessed the highest levels of heterozygosity in the SWIO area. Among the three archipelagos, the Comoros Archipelago displayed lower heterozygosity (Table 2). *F*_IS_ ranged from -0.082 to 0.110 at La Réunion sites and from -0.118 to 0.059 on Mauritius. Only 21 sites among the 127 for La Réunion and two of the 31 for Mauritius showed significant departures from Hardy-Weinberg equilibrium. When considering island- or nationwide populations, significant disequilibrium was detected for La Réunion (*F*_IS_ = 0.015), Mauritius (*F*_IS_ = 0.067), Madagascar (*F*_IS_ = 0.055), Zimbabwe (*F*_IS_ = 0.056), and South Africa (*F*_IS_ = 0.015). Significant departures from Hardy-Weinberg equilibrium were detected in France (*F*_IS_ = 0.126) and Germany (*F*_IS_ = 0.107) (see Fig 1 for relative geographic distance).

### Microsatellites detected population structure in the native continental range

Preliminary pairwise *F*_ST_ analysis between sites from the same continental countries are shown in S3 Table. Significant population differentiation was detected among African and European populations, but also among European pairs. The independent STRUCTURE analysis carried only on continental individuals suggested that the best model was K = 3 genetic clusters (S5A Fig) with a ΔK = 1959.5 (S5B and S5C Fig). Observed population structure within Africa and Europe matched earlier descriptions [12, 16, 64, 65] and expected distribution lineages (Fig 1).

### One genetic cluster exists in La Réunion, but Mauritius has two

**Fig 4:**
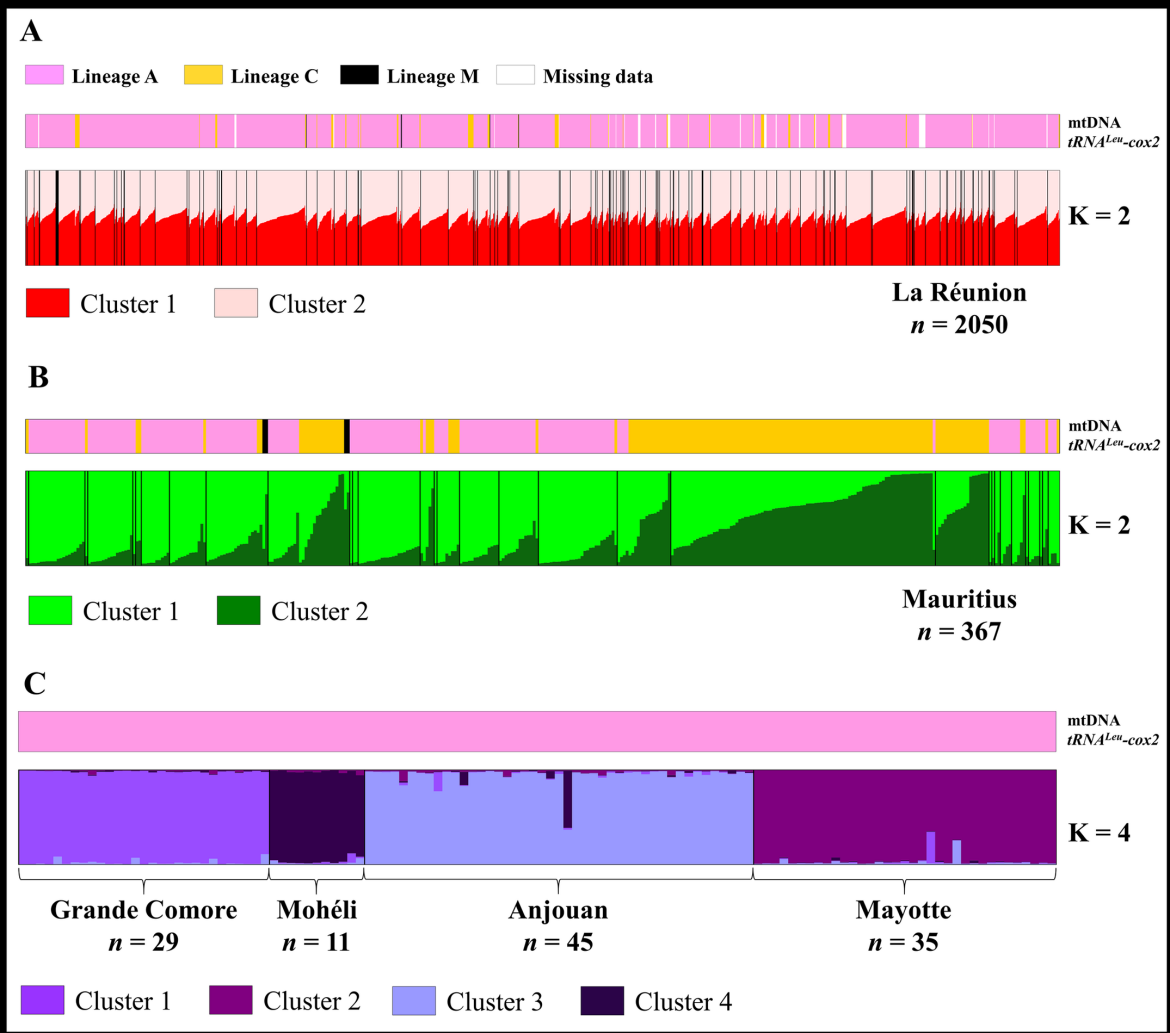
Different structuration patterns at A) La Réunion, B) Mauritius, and C) Comoros Archipelago populations in regard to maternal origin. STRUCTURE bar plots based on 14 microsatellite loci. For each optimal K model, individual probability of assignment to a genetic cluster is indicated by the height of the bar. In La Réunion and Mauritius, sites are separated by black lines and are ordered from REU001 to REU127, and MUS01 to MUS31. For the Comoros Archipelago, only islands are delimited by black lines. Individual evolutionary lineage identification based on tRNA^Leu^-cox2 intergenic region *DraI* test is presented at the top.

In La Réunion, low pairwise *F*_ST_ values between sites (from 0.004 to 0.034) were detected, but only 48 pairs out of 3,655 (considering *n* ≥ 5) were significantly different (P < 0.00037). Among these significant pairs, 25 involved two sites from remote environments located in a geological cirque (REU126 and 124). Bayesian clustering (STRUCTURE and INSTRUCT) and multivariate methods detected no genetic structuration among honey bee samples from La Réunion (*N* = 2,050) (Fig 4A). Indeed when increasing the number of assumed genetic clusters (K), an individual had similar probability of being assigned to any group (S6 Fig).

On the other hand, pairwise *F*_*ST*_ values reached much higher levels in Mauritius (-0.022 to 0.201) and 32 pairs out of the 120 were significant, including 19 involving MUS20 and 21 (same beekeeper) paired with other apiaries (P < 0.00048) (more details in S4 Table). The STRUCTURE model considering two clusters was the most likely (ΔK = 753.2) [58] (S7 Fig). When comparing nuclear diversity to mtDNA and location data, one might see that structuration was most likely linked to the presence of African or European lineage in each sampling site from Mauritius (Fig 4B). The pattern also indicated presence of “hybrid” as some individuals presenting African lineage mtDNA haplotypes were assigned (through a gradient of probabilities) to the same nuclear cluster as European C lineage individuals. The reciprocal situation was also true. Considering a probability threshold of 50%, 70.3% of the colonies from Mauritius were assigned to cluster 1 and the rest of cluster 2 (Fig 4B). Among the 109 colonies assigned to cluster 2, i) 105 showed mtDNA tRNA^Leu^-cox2 characteristic of the European C lineage and ii) 83 of these colonies belonged to sites MUS20 and 21 (same beekeeper, Fig 4B).

### Honey bee populations from the Comoros Archipelago are structured by island

**Fig 5:**
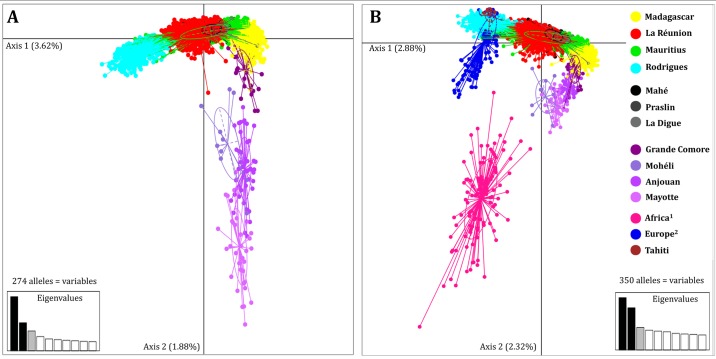
PCA of honey bee colony multilocus genotypes show population differentiation within the southwest Indian Ocean and between island and continental areas. A) PCA based on 4,125 individual multilocus genotypes from the 11 studied southwest Indian Ocean islands. B) PCA based on 4,388 individual multilocus genotypes from SWIO, continental African, European and Tahiti populations and for axis 1 and 2. Both PCAs were based on 14 microsatellites (A: 274 alleles = variables, B: 350 variables). Inertia for each axis is indicated. Each circle represents one individual and whereas individual islands and the African and European groups are distinguished by color. Africa^1^: Egypt, Senegal, São Tomé Island, Chad, Central African Republic, Cameroon, Gabon, Uganda, Malawi, Tanzania, Zanzibar, Zimbabwe, Mozambique and South Africa. Europe^2^: Switzerland, Germany, Italy, Greece, France, Spain and Portugal.

Pairwise differentiation test using *F*_ST_ values within Grande Comore, Mohéli, Anjouan, and Mayotte islands could not be performed due to limited sample sizes. Nonetheless, differentiation indices among islands of the archipelago were low, but significantly differentiated (Table 3). Mohéli was not significantly differentiated from all other populations, which might reflect the smaller sample size in the SWIO (*N* = 11) associated with the highest null allele frequency (A_null_ = 8.3%). Genetic differentiation among the Comoros islands was even visible with the distinction of four groups on PCA (Fig 5) supported by best model estimated by Bayesian clustering method (ΔK4 = 532.1, Fig 4C). At K = 2, individuals from Grande Comore and Mohéli were clustered together while Anjouan and Mayotte were assigned to the same genetic group (except one individual in Anjouan) (S8 Fig). At K = 3, individuals sampled from Anjouan were all assigned to a distinct genetic cluster. Finally, at K = 4, an intra-archipelago structure emerged as each of the four islands possessed a private genetic cluster with an exception for one individual from Anjouan (60.7% to cluster Mohéli and 36.7% to cluster Anjouan).

**Table 3:** Pairwise *F*_ST_ values among SWIO islands, and populations of African and European countries (*N* ≥ 5), based on 14 microsatellites. For each island or country, individuals from all sample sites were pooled, except for the three sites in France. Non-significant *F*_ST_ values are indicated in **bold** (after Bonferroni corrections with P < 0.000084).

	**MDG**	**REU**	**MUS**	**ROD**	**MAH**	**PRA**	**DIG**	**GCO**	**MOH**	**ANJ**	**MYT**	**STP**	**CAF**	**CMR**	**MWI**	**TZA**	**ZWE**	**ZAF**	**DEU**	**ITA**	**GRC**	**FRA01**	**FRA02**	**FRA03**	**PRT**		**0.00**
**REU**	0.14																										
**MUS**	0.11	0.04																									**0.10**
**ROD**	0.36	0.11	0.19																								
**MAH**	0.16	0.04	0.03	0.18																							**0.20**
**PRA**	0.16	0.07	0.05	0.20	0.05																						
**DIG**	0.14	0.07	0.04	0.23	0.05	0.01																					**0.30**
**GCO**	0.12	0.16	0.15	0.30	0.20	0.21	0.22																				
**MOH**	**0.25**	**0.15**	**0.18**	**0.25**	**0.19**	**0.24**	**0.25**	**0.17**																			**0.40**
**ANJ**	0.25	0.19	0.22	0.30	0.22	0.26	0.26	0.17	**0.14**																		
**MYT**	0.36	0.25	0.29	0.33	0.30	0.33	0.34	0.29	**0.23**	0.18																	**0.50**
**STP**	0.46	0.27	0.33	0.29	0.34	0.36	0.37	0.37	**0.30**	0.34	0.38																
**CAF**	0.33	0.16	0.22	0.19	0.22	0.25	0.26	0.23	**0.13**	0.22	0.27	0.12															**0.60**
**CMR**	0.38	0.17	0.25	0.20	0.25	0.29	0.31	0.29	**0.14**	0.27	0.31	**0.12**	**0.00**														
**MWI**	0.38	0.19	0.25	0.20	0.26	0.29	0.30	0.28	**0.16**	0.26	0.29	**0.11**	**0.01**	**0.00**													
**TZA**	0.34	0.16	0.21	0.20	0.22	0.24	0.25	0.23	**0.13**	0.22	0.25	0.14	**0.01**	**0.01**	**0.01**												
**ZWE**	0.34	0.16	0.22	0.20	0.22	0.25	0.26	0.24	**0.13**	0.22	0.26	0.13	**0.01**	**0.00**	**0.01**	**0.00**											
**ZAF**	0.34	0.16	0.22	0.18	0.22	0.24	0.24	0.22	**0.14**	0.22	0.24	0.12	**0.01**	**0.01**	**0.01**	**0.01**	**0.01**										
**DEU**	0.46	0.13	0.27	0.07	0.24	0.28	0.32	0.42	**0.30**	0.38	0.43	0.33	0.20	**0.19**	**0.23**	0.19	0.20	0.18									
**ITA**	0.49	0.17	0.30	0.08	0.29	0.32	0.36	0.47	**0.39**	0.44	0.48	0.42	0.32	0.33	0.35	0.32	0.32	0.29	0.05								
**GRC**	0.49	0.17	0.32	0.14	0.28	0.34	0.37	0.47	**0.35**	0.41	0.47	0.37	**0.23**	**0.24**	**0.27**	0.24	0.24	0.22	0.10	0.15							
**FRA01**	0.48	0.30	0.34	0.27	0.37	0.39	0.41	0.43	**0.38**	0.40	0.45	0.33	0.24	0.25	0.23	0.22	0.22	0.19	0.38	0.45	**0.48**						
**FRA02**	0.46	0.27	0.31	0.24	0.34	0.36	0.37	0.38	**0.33**	0.36	0.40	0.28	0.20	0.20	0.18	0.18	0.18	0.15	0.31	0.40	0.41	0.03					
**FRA03**	0.41	0.12	0.23	0.09	0.23	0.27	0.30	0.36	**0.24**	0.32	0.37	0.25	0.13	**0.12**	**0.15**	0.13	0.14	0.12	0.06	0.15	0.13	0.26	0.22				
**PRT**	0.52	0.33	0.38	0.30	0.41	0.42	0.44	0.46	**0.40**	0.43	0.46	0.34	0.24	**0.26**	**0.24**	0.22	0.22	0.20	0.40	0.47	**0.51**	0.09	0.07	0.30			
**TAH**	0.51	0.19	0.32	0.10	0.30	0.34	0.38	0.51	**0.42**	0.46	0.51	0.44	0.32	**0.34**	0.35	0.31	0.31	0.28	0.08	**0.01**	**0.18**	0.49	0.42	0.17	0.51		

MDG: Madagascar, REU: La Réunion, MUS: Mauritius, ROD: Rodrigues, MAH: Mahé, PRA: Praslin, DIG: La Digue, GCO: Grande Comore, MOH: Mohéli, ANJ: Anjouan, MYT: Mayotte, STP: São Tomé, CAF: Central African Republic, CMR: Cameroon, MWI: Malawi, TZA: Tanzania, ZWE: Zimbabwe, ZAF: South Africa, DEU: Germany, ITA: Italy, GRC: Greece, FRA01-03: France and PRT: Portugal.

### Global dataset structure analysis showed genetic distinction between SWIO and outgroup populations

**Fig 6:**
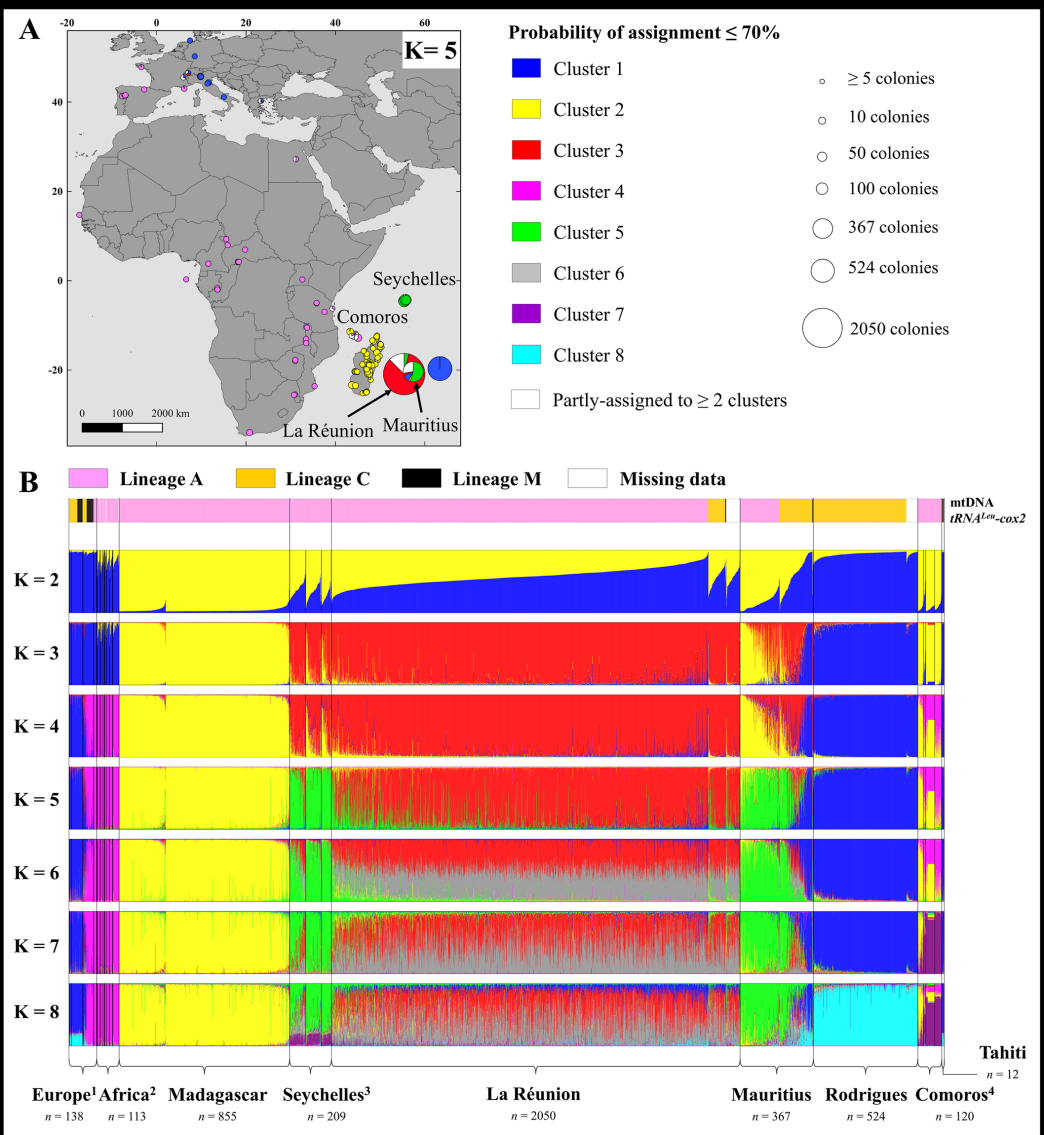
Western honey bee populations from southwest Indian Ocean islands are genetically structured both at global and local scales. A) Geographic distribution of the five genetic clusters (K = 5) using an assignment probability threshold ≥ 70%. B) Global STRUCTURE bar plots are presented for K = 2 to 8, based on 4,388 honey bees. No prior location information was given to the Bayesian clustering analysis. SWIO islands are separated by black lines and delimitation for continental outgroups is by country. Maternal origin of each individual (evolutionary lineage A, C, or M) as determined by the tRNA^Leu^-cox2 *DraI* test is presented in the upper bar plots.

The complexity of honey bee phylogeography in the SWIO was represented in the Fig 6 by STRUCTURE bar plots and distribution of the detected genetic clusters. For the Bayesian method, the uppermost value of K (K = 2) was not coherent with results of other analyses such as PCA (Fig 5B), DAPC, or with STRUCTURE runs computed on each island. To avoid interpretation problems, the choice of K followed recommendation for such case [66] by considering the Ln(K) standard deviation (S9 Fig). DAPC method gave similar results that are available in supplemental S10 and S11 Figs. Here, we concluded that K = 5 is the most appropriate model based upon the present sampling.

Considering a probability threshold of 70%, at K = 5, colonies sharing same African mtDNA haplotypes in Madagascar, La Réunion, Mauritius, and Seychelles were assigned to different genetic clusters (Fig 6B). At K = 5, Mascarenes Archipelago colonies were grouped by island i) 84.0% of La Réunion in *red* cluster 3, ii) 54.8% of Mauritius in *green* cluster 5 and iii) 99.6% of Rodrigues colonies assigned to *blue* cluster 1. As SWIO reference, 99.4% of Madagascar colonies clustered to *yellow* cluster 2. Mauritius shared a nuclear genetic diversity (alleles) with Seychelles colonies mostly assigned to the same cluster (96.4% to *green* cluster 5 for Mahé, Praslin, La Digue confounded). Compared to all other populations, Mauritius presented the most mixed population with four clusters (Fig 6). Global STRUCTURE analysis showed that one Mauritius cluster is highly similar to individuals from Europe and Rodrigues. Finally, the Comoros Archipelago presented an interesting mixture of cluster 4 (*pink*) derived mainly from continental Africa and cluster 2 mainly found in Madagascar. A geographic paradox appeared as 100% of the colonies from the most distant island to Madagascar, Grande Comore, was assigned to the same cluster. This SWIO island structure was also supported by significant differentiation estimated by pairwise significant *F*_*ST*_ values, but still lower than those of the continents (Table 3). In addition, PCA (Fig 5) showed that SWIO island point scatters were all distinct apart for the case of Mauritius overlapping with several populations.

The global population structure view and the progressive increment of K allow to show multiple conclusions: i) Madagascar honey bees were genetically distinct from continental African colonies, ii) La Réunion bees differentiated early from Madagascar genetic group, iii) Mauritius and Seychelles bees share a genetic background with La Réunion, but were still differentiated, iv) the second intra-cluster found on Mauritius was associated to one European cluster, v) Comoros Archipelago bees share a genetic background with continental African populations and Madagascar, but still formed a different population, and vi) Rodrigues shares a genetic background with European populations, yet formed a new population, unlike Tahiti.

## Discussion

This study shows that honey bee (*A*. *mellifera*) populations established in the Mascarenes and Comoros archipelagos present high nuclear microsatellite polymorphism for insular populations. Despite having generally similar African mtDNA backgrounds, different levels of nuclear genetic differentiation were apparent within and between archipelagos. No genetic structure was found at La Réunion, while in Mauritius, genetic data indicated the coexistence of two clusters. Both populations have a close relationship to Madagascar honey bees rather than to native African continental populations. Ongoing hybridization between African and European lineages was evident in Mauritius.

In the SWIO, several insular populations occur, and each one possesses a singular genetic pattern in terms of evolutionary lineages and nuclear genetic diversity and structure. Such a complex evolutionary pattern will need to be broken down for each island, in the future.

### The Comoros Archipelago as a contact area between Africa and Madagascar

Comoros Archipelago honey bee colonies analyzed are exclusively descended from African lineages, and more particularly the mitochondrial A_I_ sub-lineage observed both in Madagascar and the main part of Africa [15, 37, 40]. The previous sequencing of two mtDNA non-coding and coding regions showed that Comoros honey bees mainly share a common haplotype with *A*. *m*. *unicolor* in Madagascar, but also exhibit a private haplotype [37]. These colonies also display shared nuclear alleles with Madagascar and African continental populations, putting them in an intermediate position. The Comoros Archipelago is equidistant between Africa and Madagascar (300 km) and could represent an exchange pathway between landmasses.

Each island possesses specific genetic clusters clearly differentiated from the closest neighboring island. Such a pattern is not surprising for insular honey bee populations, as it has been previously reported for the Seychelles [38] and other archipelagos, such as the Canary Islands [67], Madeira Islands, and the Azores [68, 69]. The four Comoros islands are separated by an oceanic barrier of approximately 40 km (Grande Comore—Mohéli) to 190 km (Grande Comore—Mayotte), which may have been sufficient to restrict gene flow among the islands. Genetic structure within the archipelago also suggests no recent human-mediated introductions of honey bees from neighboring islands. Unlike the Seychelles Archipelago, beekeeping is not developed in the Comoros Archipelago, so human-mediated exchanges are unlikely to have occurred among the four islands.

Paradoxically, Grande Comore, the closest island to the East African coast, was the most genetically similar to Madagascar. The other three islands indicate progressive colonization and gene flow from the African coast and Madagascar, likely via island hopping, as observed for chameleons [70]. On Grande Comore, the unexpected highest proportion of individuals assigned to the Madagascar cluster, probably indicates a recent unreported human introduction.

#### The African honey bee genetic background predominates in La Réunion

La Réunion was dominated by *A*. *m*. *unicolor* colonies (95.2%), but mtDNA sequences characteristic of *A*. *m*. *mellifera*, *A*. *m*. *carnica*, and *A*. *m*. *ligustica* were also found. However, if multivariate analysis indicated that this population was similar to, but distinct from Madagascar, it also showed a certain proximity to European colonies. The presence of European honey bee colonies confirmed previous reports stating that introduction of exotic subspecies occurred before import prohibitions established in 1982 [71]. Unlike Rodrigues or Mauritius, from the same archipelago, no honey bees with European mtDNA differed from African colonies in their nuclear identities. Conversely no individual with African mtDNA clustered with European colonies on the basis of nuclear genetic diversity. Such patterns could result from low importation levels compared to the large African pool preexistent in La Réunion. In addition, asymmetric introgression in favor of African over European lineages is a known phenomenon in honey bees defined as Africanized [72–76]. Differences in sperm-related genes were proposed to explain this reproductive advantage of African vs. European honey bees [12].

Despite the topology of La Réunion, reaching an elevation of 3069 m [77], no genetic structure was observed across the island. This indicated that gene flow is sufficient to maintain genetic homogeneity. Exchanges between distant locations may have been facilitated by intra-island beekeeping movements following resource cycles. Beekeepers from La Réunion move their hives two or three times per year from the lowlands to the highlands, and from the humid east coast to the drier west coast, following the availability of flowering plants. Homogenization of the genetic pool by such practices has already been described in continental honey bee populations [78].

Interestingly, La Réunion has the highest proportions of African colonies and undegraded native forest habitats, estimated at 25% of the original cover [77]. This difference in environmental conditions compared to Mauritius (2% original forest cover) and Rodrigues (0%) has undoubtedly influenced the established populations. Natural selective pressures exerted on La Réunion colonies may be advantageous to tropically adapted subspecies, such as *A*. *m*. *unicolor*, more than to temperate, introduced subspecies. However, it would be interesting to investigate this in wider genomic and coding regions to determine whether this adaptation is due only to the African genome. As an example, it has recently been shown that a positive selection signal appeared in Chromosome 11 with an excess of European over African ancestry which may confer an adaptive advantage to Africanized honey bee populations found in Brazil [79].

#### Admixed population and recent hybridization undergoing in Mauritius

For Mauritius, the genetic composition of honey bee populations was the most complex case among all the SWIO islands, highlighting the impact of human honey bee importation. In our sampling, similar proportions of local and exotic lineages were detected. However, one apiary highly contributed to the European frequency as a beekeeper imported a huge batch of *A*. *m*. *ligustica*-like queens [41]. As in La Réunion, European lineages present on Mauritius corroborates records stating that regular introductions were carried out [41, 80]. Nuclear analysis uncovered on-going hybridization on this island between African and European genetic groups. Mixing of these two divergent lineages was revealed by cytoplasmic disequilibrium as several individuals with *A*. *m*. *unicolor* mtDNA haplotypes were poorly differentiated and were assigned to the same genetic cluster as native European colonies, and the reverse occurred. In both mtDNA African and European colonies found in Mauritius, a continuum in nuclear assignment toward the opposite cluster suggested ongoing introgression in both directions. Such phenomena have already been reported on other European islands and between the African and European C lineage in *A*. *m*. *iberiensis* [69] and *A*. *m*. *siciliana* [81].

As at Rodrigues, C and M colonies were genetically similar to continental populations of *A*. *m*. *ligustica* and *carnica*. In Mauritius, two apiaries (MUS20 and 21) showed “pure” European colonies due to relatively recent introductions confirmed by field investigation and government reports [41]. Whether these colonies originated directly from native continental areas in Europe or were introduced from another exotic population could only be determined with wider sampling. Heavy deforestation, leaving only relictual native vegetation in Mauritius, and replacement with high proportions of exotic plants may have also influenced survival of European colonies [82, 83].

#### Genetic diversity and structure in the SWIO reflects island effects and influences of beekeeping practices

Geographic and climatic barriers have played an important role in the evolution of the Western honey bee (*A*. *mellifera*) into five lineages with up to 31 subspecies [13, 84, 85]. The divergence signal can be weak in the case of landscape continuum as for *A*. *m*. *iberiensis* [86, 87] or sharper, as in the genetic differentiation of *A*. *m*. *unicolor* from Madagascar [40]. However, a comprehensive picture of indigenous *A*. *mellifera* population histories may be difficult to develop due to the long relationship with humans and modern global transportation of honey bee colonies [20, 21]. The occurrence of a novel mitochondrial SWIO African sub-group and private haplotypes in the Mascarenes (except Rodrigues), Comoros and Seychelles suggested ancient colonization events [37]. The larger screening of the tRNA^Leu^-cox2 intergenic region done here in the SWIO populations confirmed that these insular populations are mainly of African origin. Since mtDNA indicates only maternal lineage, the nuclear genetic diversity observed confirmed that the SWIO islands (excepted Rodrigues) are more closely related to Madagascar than to any African populations. Nevertheless, the differentiation index (*F*_*ST*_), Bayesian, and multivariate analyses showed that SWIO populations present nuclear genetic diversity distinct from Madagascar. Yet this observed divergence between Madagascar and others SWIO islands was still lower than that observed between continental African A and European M and C populations.

Nuclear genetic structuration among SWIO populations could be explained by several non-exclusive hypotheses. First, to obtain such genetic differentiation levels among islands, geographic isolation by ocean barriers should have been maintained long enough to restrict the gene flow, resulting in homogenization. By comparison, the local honey bee from the Balearic Archipelago experienced introduction probably in the XVIII and XIX centuries, and the *F*_*ST*_ values among populations (0.04–0.27 [88]) were of the same order of magnitude as in the Mascarenes (*F*_*ST*_ = 0.04 to 0.19) or the Comoros (*F*_*ST*_ = 0.14 to 0.29). Evidence of gene flow between La Réunion, Mauritius, and the Seychelles Archipelago is highlighted by global Bayesian clustering analysis. This may have resulted from past connections via the exchange of colonies, possibly during the colonial period through the route to India in the XVIII century.

Secondly, genetic differentiation among islands could be due only to divergent lineages assembly. Nevertheless, in native continental areas, *F*_*ST*_ values among populations were ≥2x higher than among SWIO islands with different evolutionary lineages coexistence. In comparison, Rodrigues, which is likely from a European genetic background (mtDNA and microsatellites), showed differentiation values higher than the admixed population of Mauritius, La Réunion, or any other islands from the Seychelles or Comoros archipelagos. This could mean that the genetic diversity pool created by European colonies brought to La Réunion and Mauritius might not fully explain their differentiation from other local populations. If not European, the genetic differentiation may have resulted from colonization by or introduction of African lineages. However, Madagascar was the closest native African population the SWIO islands and none of the continental African populations showed similar proximity (congruence of all analyses: PCA, STRUCTURE, and genetic differentiation test).

At the time when *A*. *mellifera* started to diverge throughout its native range, all islands of the SWIO were completely formed, and the Mascarenes were already colonized by angiosperms. For example, the Dombeyoideae family (~ 25 to 35 Ma [89]) and the *Acacia heterophylla* species, visited by honey bees now, are believed to have reached the island around 1.4 Ma ago [90] from Hawaii. Consequently, these islands already possessed habitats suitable for generalist pollinators that require pollen and nectar for survival. Madagascar has been identified several times as a base for colonization and radiation into neighboring archipelagos, whether for flora [91–93] or fauna [94–97]. The relatively lower genetic diversity observed in the 11 islands of the SWIO compared to African continental populations suggested progressive colonization [9]. A loss of genetic diversity could be associated with founder events where only a sub-sample of African diversity reached Madagascar. After diversification on that island, a similar evolutionary process likely occurred in the Mascarene, Seychelles, and Comoros archipelagos. Despite being the potential source population for SWIO islands, Madagascar had lower levels of heterozygosity and alleles per locus than any other island. One hypothesis is that Madagascar colonies experienced bottleneck events, possibly due to loss and fragmentation of original habitats caused by deforestation [98]. A similar observation was made on African honey bee colonies, deforestation being identified as the major threat to wild African colonies [99]. A second possible explanation is that part of the high genetic diversity observed in the SWIO archipelago in regard to Madagascar is the result of admixture, which reduces the negative effects of a bottleneck [100] and has been shown to increase diversity levels in honey bee populations [23]. In all the SWIO archipelagos, hybridization occurred or is ongoing with dissimilar assemblies African A_I_-Malagasy in Comoros, African Z-Malagasy in Seychelles [38], and African-European in the Mascarenes. All these elements, combined with significant population differentiation among SWIO insular honey bee populations, seem consistent with “natural” colonization. Yet, this requires further investigation, as several factors are unknown for each of these populations, complicating interpretation.

## Conclusions

Genetic diversity and structure of honey bee populations of SWIO islands suggests ancient colonization events of *A*. *m*. *unicolor* from Madagascar to the Mascarenes and Seychelles archipelagos, old enough to detect population differentiation within the sub-lineage. The use of nuclear and mitochondrial markers uncovered the presence of exotic subspecies and different levels of hybridization with indigenous populations in the archipelagos. The numerous interactions recorded between *A*. *mellifera* and endemic species [33, 34, 36, 101–105] with some remarkably benefits [106], stress the importance of preserving this species.

Apart from its ecological role, these populations with singular genetic diversity deserve particular attention, especially against the global loss of honey bee colonies [107–109]. Now that whole-genome sequencing has become more affordable, it would be interesting to investigate the effects of hybridization between African and European lineages in the SWIO islands using a genomic approach. Such data could offer better resolution for estimating times of divergence and would allow us to better retrace the demographic history of these insular populations.

## Supporting information

S1 Fig Distribution of honey bee colony sampling sites in Madagascar, and the Seychelles and Mascarenes archipelagos. *First line and from left to right*: Geographic positions of 127 sampling sites from La Réunion, 31 from Mauritius, and 20 from Rodrigues in the Mascarenes Archipelago. *Second line and from left to right*: Geographic positions of the 81 sampling sites from Madagascar, 43 sites in the Seychelles Archipelago with 22 sites from Mahé, 16 from Praslin, and 5 sites from La Digue. *N* = Number of honey bee colonies sampled by island. Layer used for QGIS map is Open Street Map. Sampling † from (39), * (40), ˟° (38).
(TIF)

S2 Fig Distribution of honey bee colony sampling sites in the four islands of the Comoros Archipelago. (TIF)

S3 Fig Sampling effort represented by allele accumulation curves for 14 microsatellite loci in La Réunion, Mauritius, and the Comoros Islands, compared to other insular and continental populations. (A) Overall sampling size scale and (B) comparative lower scale. Only the three largest continental populations of Italy, France, and South Africa are represented to increase readability.
(TIF)

S4 Fig Mean number of alleles per locus (14 microsatellites) within La Réunion and Mauritius (sites with *n* ≥ 5). (TIF)

S5 Fig European and African samples are good representative outgroups for native honey bee populations structured using the distribution of mtDNA lineages. **A)** STRUCTURE bar plots (K = 2 to 5) for 263 honey bee colonies sampled in Africa and Europe, inferred from 14 microsatellite loci. Each vertical line represents the posterior assignment probability of a single individual to one or more genetic clusters (one color). Sites are separated by black lines. Maternal origin for each individual (evolutionary lineage A, C or M) defined by the *DraI* test on the COI-COII intergenic region is presented at the top. **B)** Average likelihood of runs in STRUCTURE L(K) along with number of K clusters for African and European sites. **C)** ΔK, estimator of the optimal number of clusters (K) according to Evanno et al. (58). The two graphs were created using Structure Harvester (61).
(TIF)

S6 Fig Absence of population structure in the 2,050 honey bee colonies sampled from 127 sites at La Réunion, based on 14 microsatellite loci. A) STRUCTURE bar plots at K = 2, B) Average likelihood of runs in STRUCTURE L(K) along with number of clusters (K) for La Réunion. C) ΔK, estimator of the optimal number of clusters (K) according to Evanno et al. (58).
(TIF)

S7 Fig Coexistence of two genetic clusters and hybrid honey bees in Mauritius (*N* = 367), based on 14 microsatellite loci. A) STRUCTURE bar plots at K = 2 and 3. Sites are separated by black lines and are ordered from MUS01 to 31. Maternal origin (top) for each individual (evolutionary lineage, A, C, or M) defined by the *DraI* test on the COI-COII intergenic region. **B)** Average likelihood of runs in STRUCTURE L(K) with the number of K clusters for Mauritius. **C)** ΔK, estimator of the optimal number of clusters (K) according to Evanno et al. (58).
(TIF)

S8 Fig Genetic structure of honey bee populations from islands in the Comoros Archipelago, inferred from 14 *loci* microsatellites. A) STRUCTURE bar plots from K = 2 to 5. All colonies had haplotypes from the COI-COII intergenic region characteristic of the African evolutionary lineage. B) Average likelihood of runs in STRUCTURE L(K) along with number of K clusters for Comoros Archipelago. C) ΔK, estimator of the optimal number of clusters (K) according to Evanno et al. (58).
(TIF)

S9 Fig A) Average likelihood of runs in STRUCTURE L(K) along with number of K clusters for global STRUCTURE based on 4,388 honey bees Comoros Archipelago (Fig 6). B) ΔK, estimator of the optimal number of clusters (K) according to Evanno et al. (58).
(TIF)

S10 Fig DAPC barplots of the Western honey bee populations from southwest Indian Ocean islands at global scale. DAPC bar plots are presented for K = 3 to 8, based on 4,388 honey bees.
(TIF)

S11 Fig Relationship among the different genetic clusters computed using DAPC approach on 4,388 samples of the Western honey bee. Colors of the different clusters correspond to the S10 Fig.
(TIF)

S1 Table Complete sample database, including sample IDs, location coordinates, mtDNA COI-COII *DraI* profiles, and multi-locus genotypes determined at 14 microsatellite loci. (XLSX)

S2 Table Mitochondrial COI-COII intergenic region diversity (based on *DraI* restriction profiles) and nuclear diversity indices for each SWIO, African, and European sampling site. N: number of colonies per site; NA_COI-COII_: number of individuals with missing COI-COII data, N_all_: mean number of alleles; H_nb_ and H_obs_: unbiased expected and observed heterozygosity, respectively; *F*_IS_ (* significant at P < 0.05) and A_null_: mean allele null frequency.
(DOCX)

S3 Table Pairwise *F*_ST_ values among sites from Zimbabwe (ZWE), France (FRA), and Italy (ITA) with N ≥ 5, based on 14 microsatellites. After Bonferroni corrections, permutations tests were only significant among French sites (in bold P < 0.000549). Colors as in Table 3.
(DOCX)

S4 Table Pairwise *F*_ST_ values among sites at Mauritius Island with N ≥ 5 based on 14 microsatellites. Statistical significance for the permutation tests after Bonferroni corrections is indicated in bold (P < 0.000476). Colors as in Table 3.
(DOCX)

## Abbreviations

cox2: Cytochrome oxidase subunit II gene
Ma: millions of years ago
mtDNA: mitochondrial DNA
ND2: NADH-dehydrogenase subunit 2 gene
PCR-RFLP: Polymerase Chain Reaction—Restriction Fragment Length Polymorphism
SWIO: southwest Indian Ocean
tRNA^Leu^-: Transfer RNA Leucine
