# Supplementary figures and images for: Genetic diversity and differentiation among insular honey bee populations in the southwest Indian Ocean likely reflect old geographical isolation and modern introductions
Source: PLoS One. 2017 Dec 27;12(12):e0189234. doi: 10.1371/journal.pone.0189234 (PMC5744932; doi:10.1371/journal.pone.0189234)

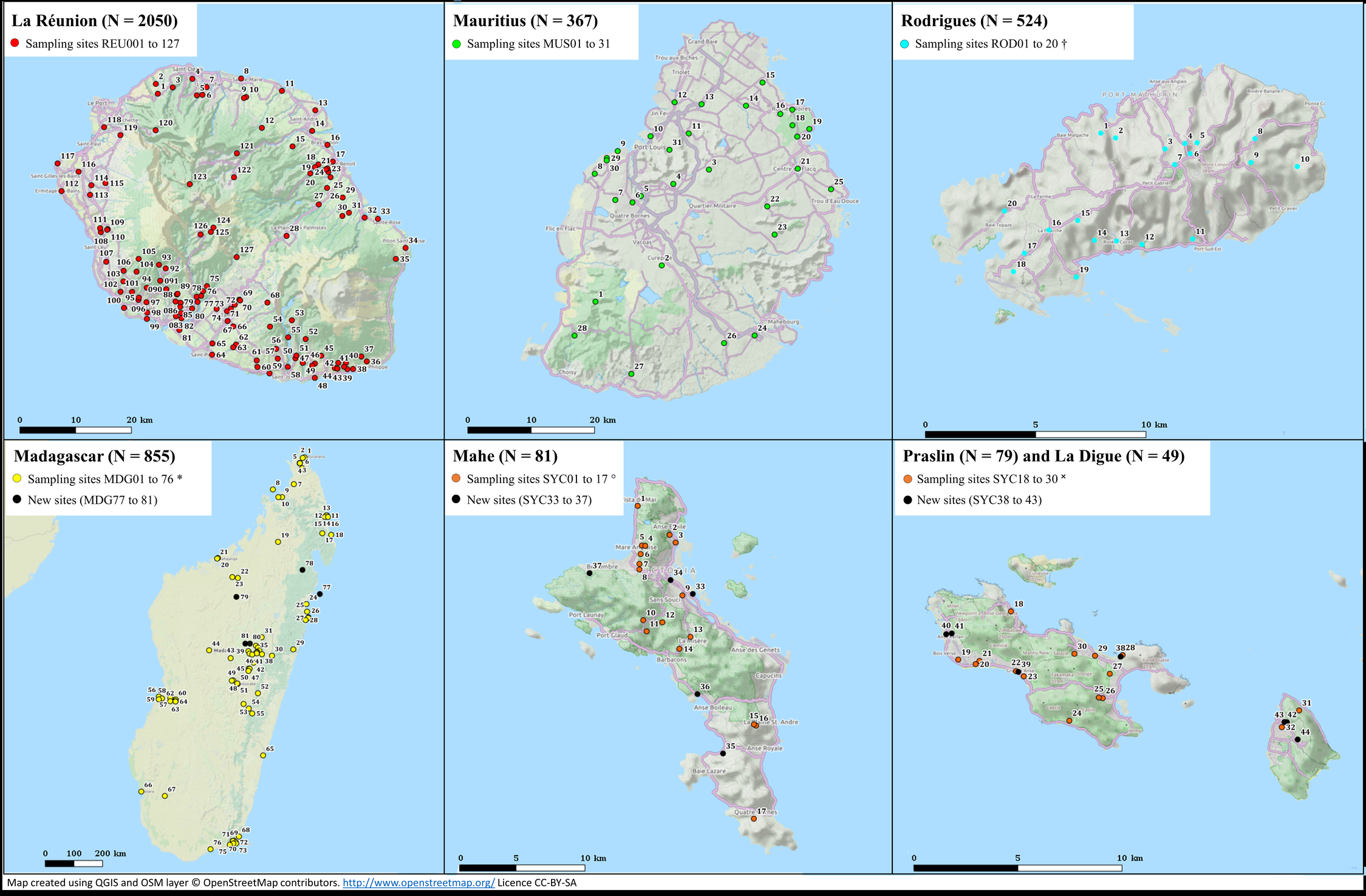

Supplement: S1 Fig — First line and from left to right: Geographic positions of 127 sampling sites from La Réunion, 31 from Mauritius, and 20 from Rodrigues in the Mascarenes Archipelago. Second line and from left to right: Geographic positions of the 81 sampling sites from Madagascar, 43 sites in the Seychelles Archipelago with 22 sites from Mahé, 16 from Praslin, and 5 sites from La Digue. N = Number of honey bee colonies sampled by island. Layer used for QGIS map is Open Street Map. Sampling † from (39), * (40), ˟° (38). (TIF) [file pone.0189234.s001.tif]

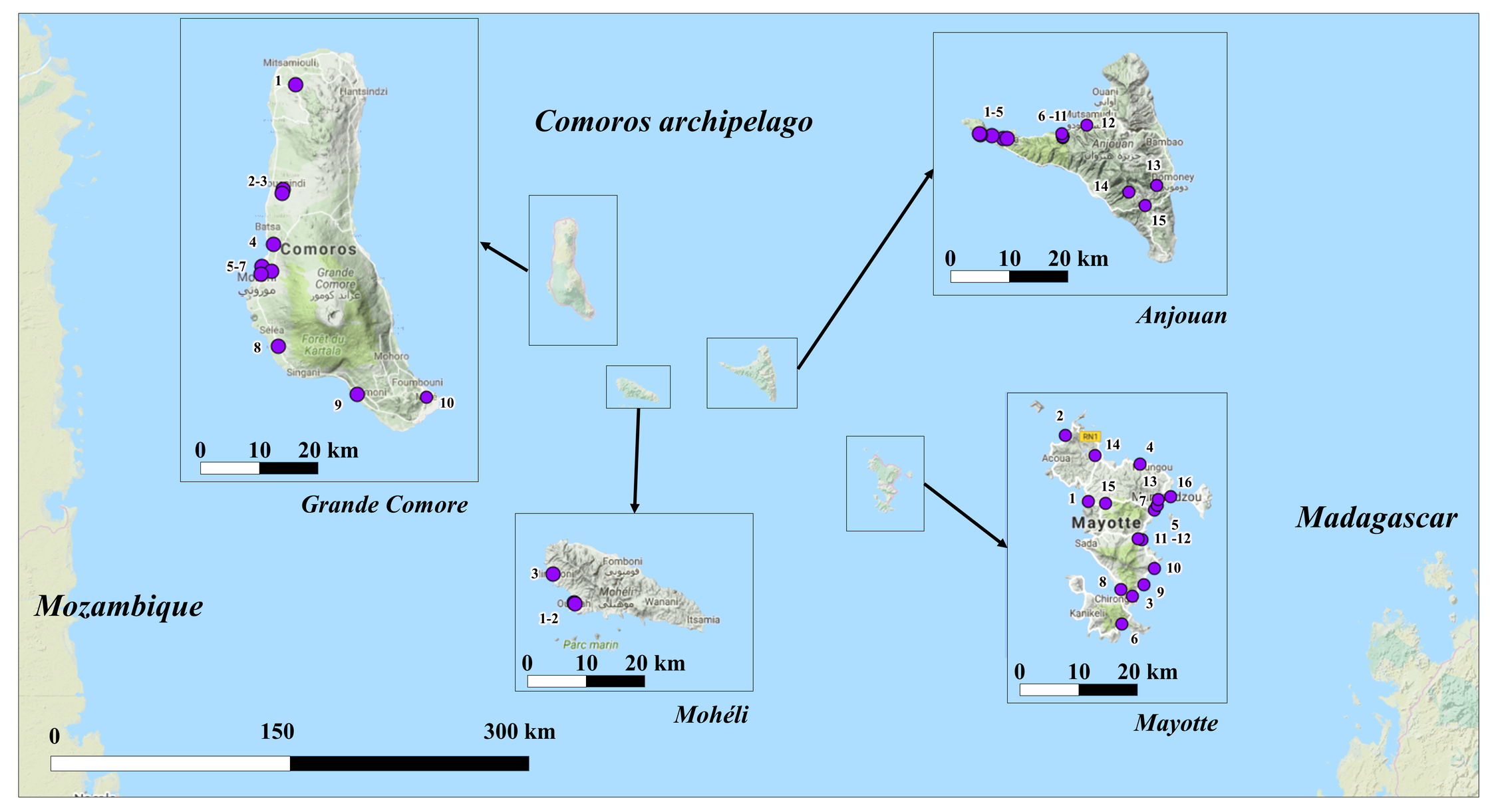

Supplement: S2 Fig — (TIF) [file pone.0189234.s002.tif]

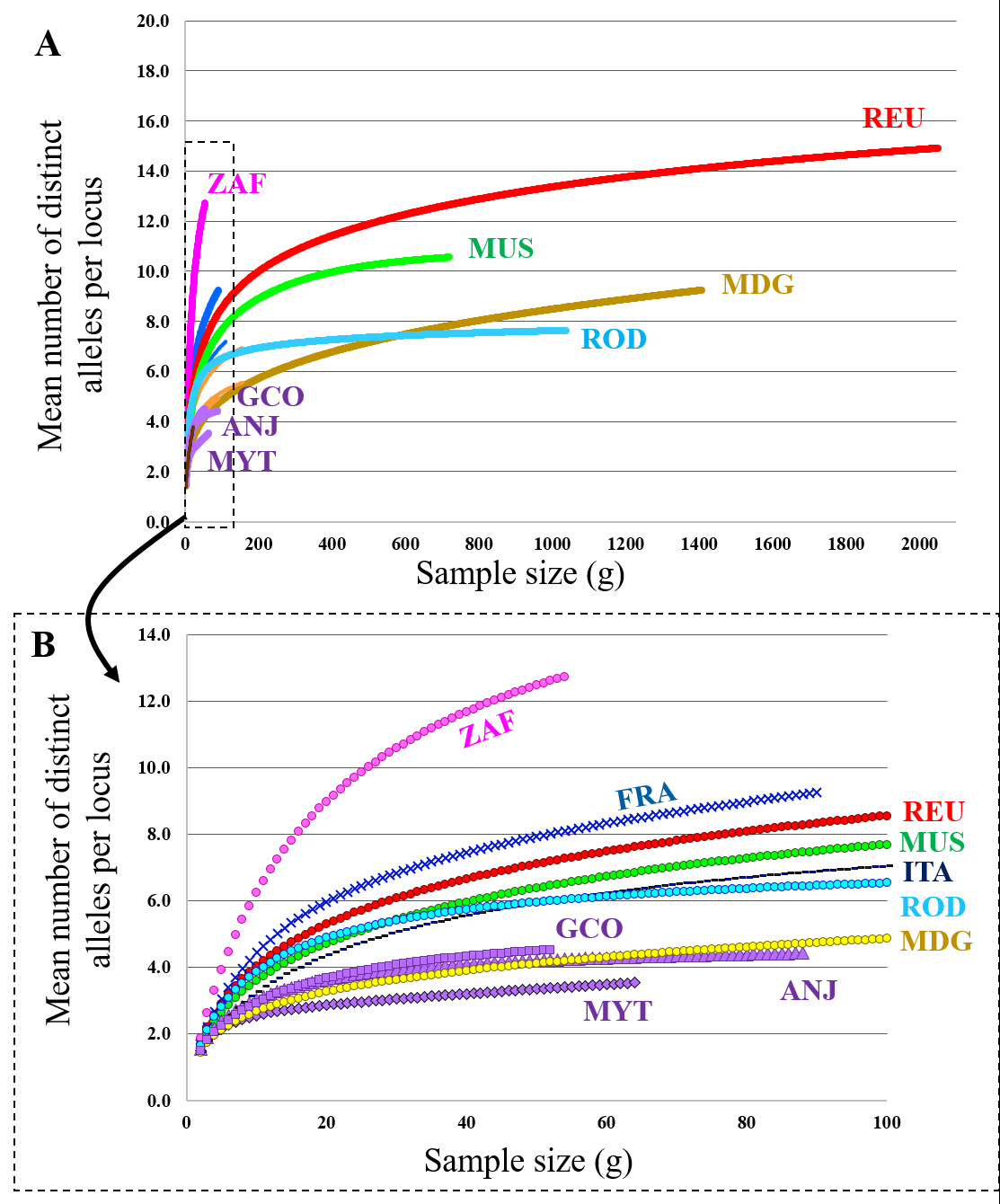

Supplement: S3 Fig — (A) Overall sampling size scale and (B) comparative lower scale. Only the three largest continental populations of Italy, France, and South Africa are represented to increase readability. (TIF) [file pone.0189234.s003.tif]

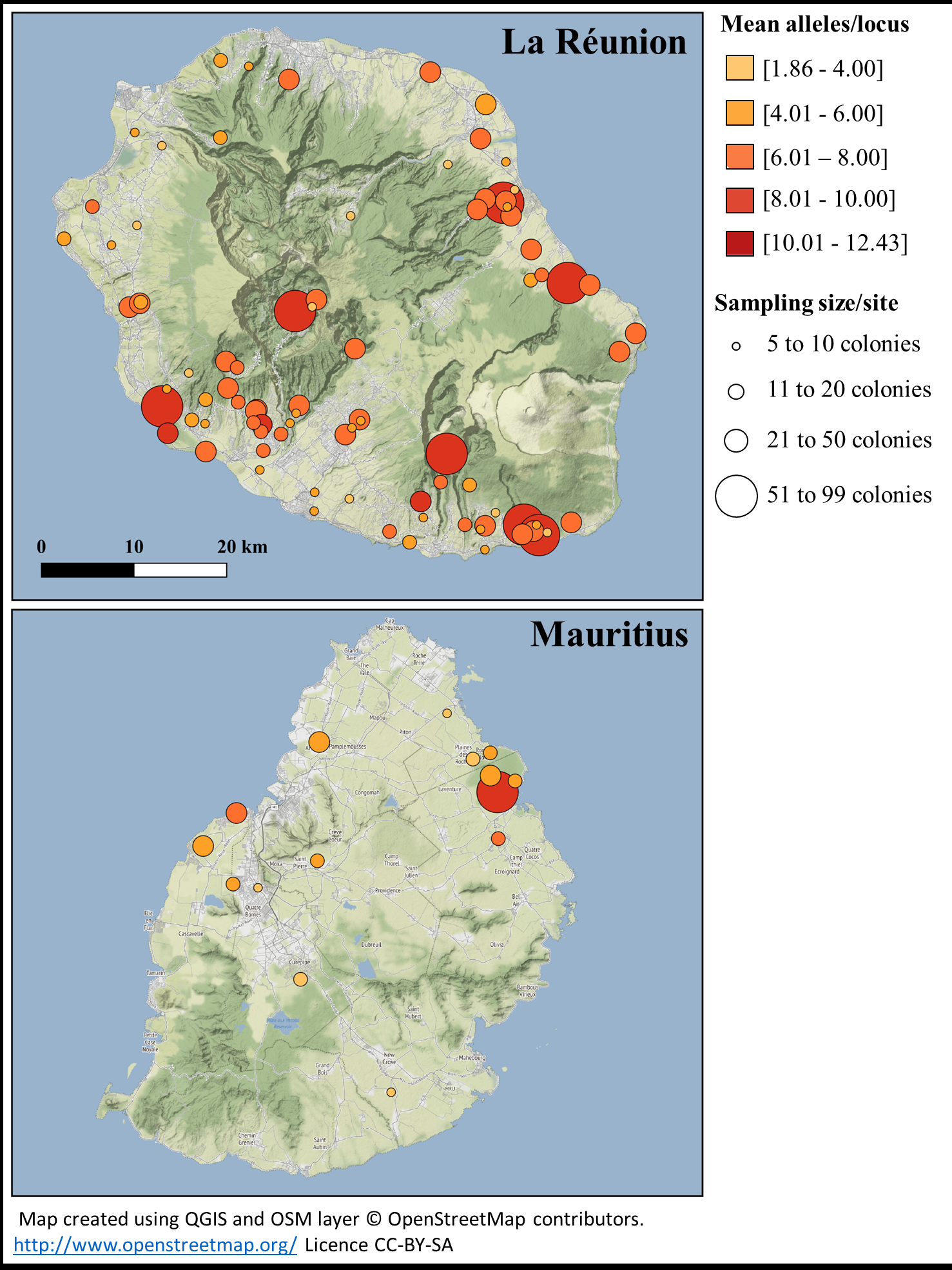

Supplement: S4 Fig — (TIF) [file pone.0189234.s004.tif]

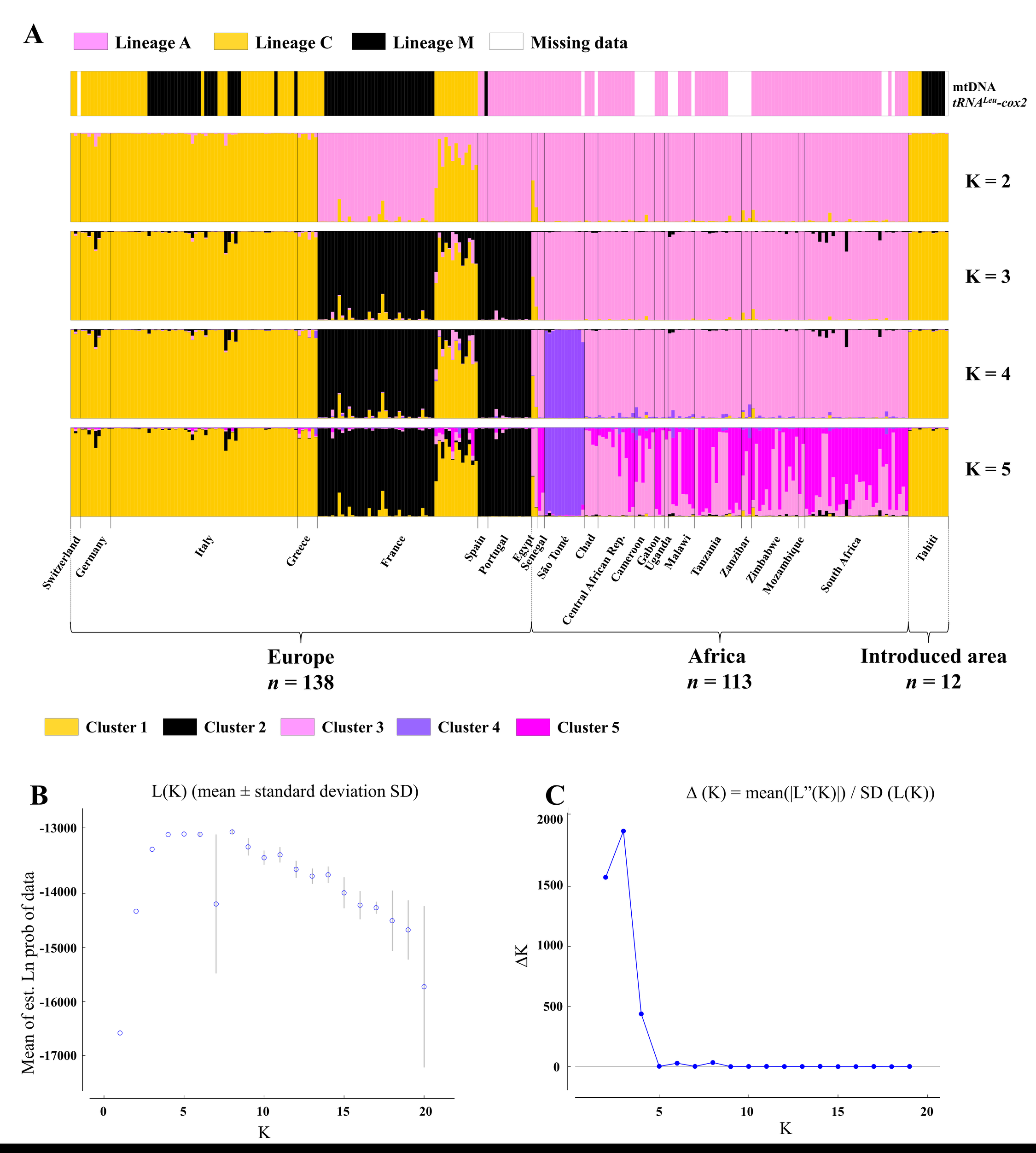

Supplement: S5 Fig — A) STRUCTURE bar plots (K = 2 to 5) for 263 honey bee colonies sampled in Africa and Europe, inferred from 14 microsatellite loci. Each vertical line represents the posterior assignment probability of a single individual to one or more genetic clusters (one color). Sites are separated by black lines. Maternal origin for each individual (evolutionary lineage A, C or M) defined by the DraI test on the COI-COII intergenic region is presented at the top. B) Average likelihood of runs in STRUCTURE L(K) along with number of K clusters for African and European sites. C) ΔK, estimator of the optimal number of clusters (K) according to Evanno et al. (58). The two graphs were created using Structure Harvester (61). (TIF) [file pone.0189234.s005.tif]

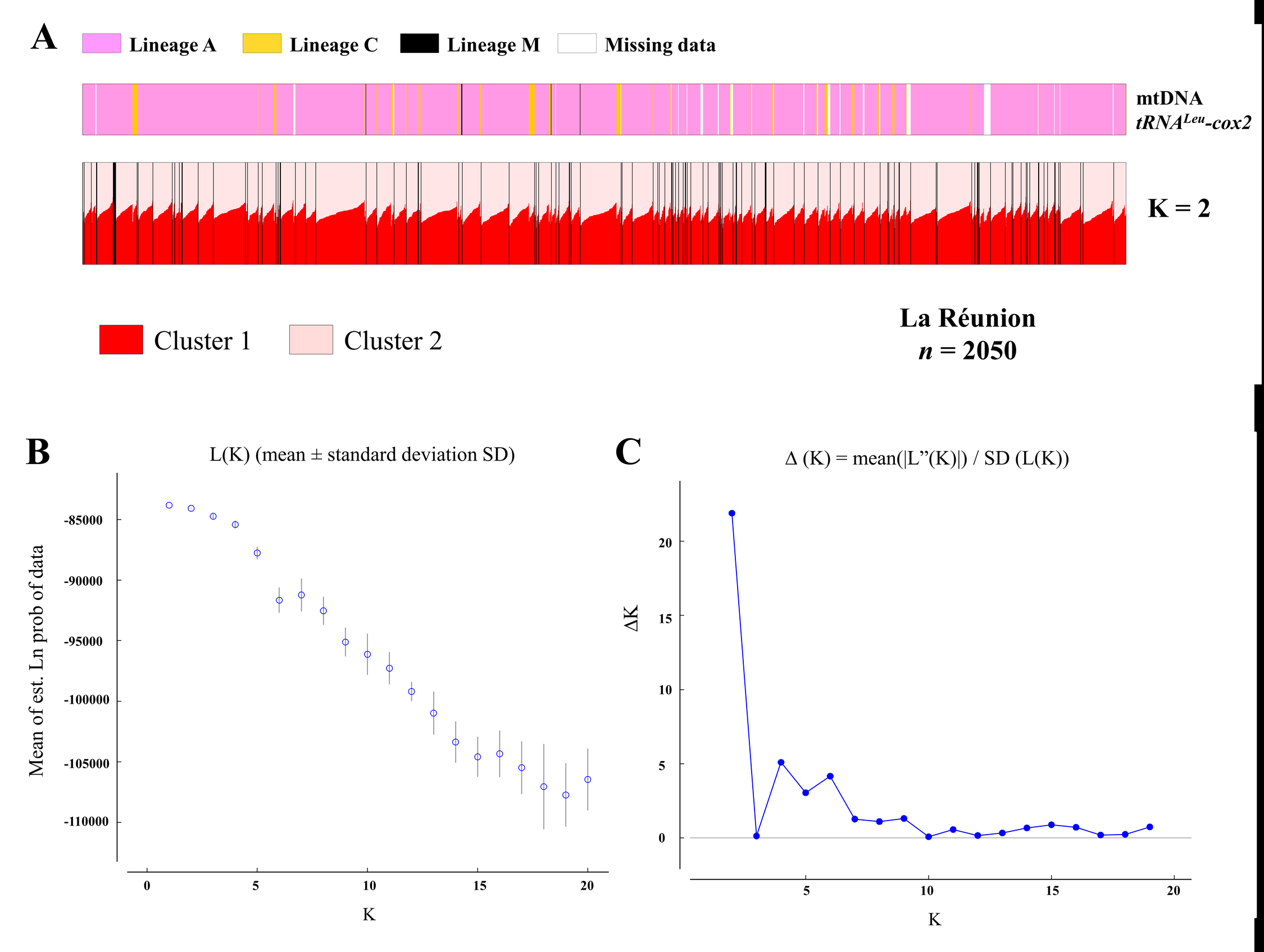

Supplement: S6 Fig — A) STRUCTURE bar plots at K = 2, B) Average likelihood of runs in STRUCTURE L(K) along with number of clusters (K) for La Réunion. C) ΔK, estimator of the optimal number of clusters (K) according to Evanno et al. (58). (TIF) [file pone.0189234.s006.tif]

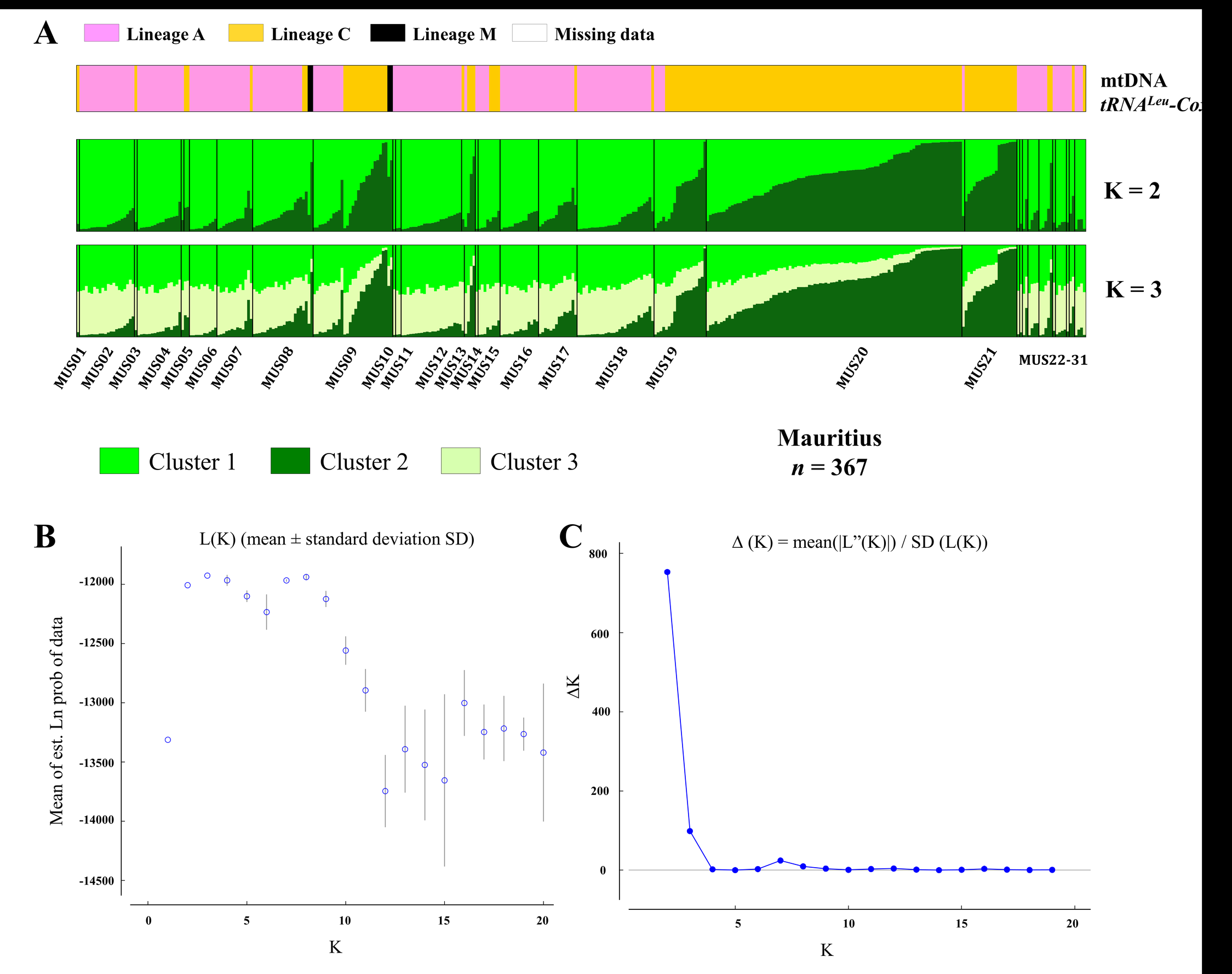

Supplement: S7 Fig — A) STRUCTURE bar plots at K = 2 and 3. Sites are separated by black lines and are ordered from MUS01 to 31. Maternal origin (top) for each individual (evolutionary lineage, A, C, or M) defined by the DraI test on the COI-COII intergenic region. B) Average likelihood of runs in STRUCTURE L(K) with the number of K clusters for Mauritius. C) ΔK, estimator of the optimal number of clusters (K) according to Evanno et al. (58). (TIF) [file pone.0189234.s007.tif]

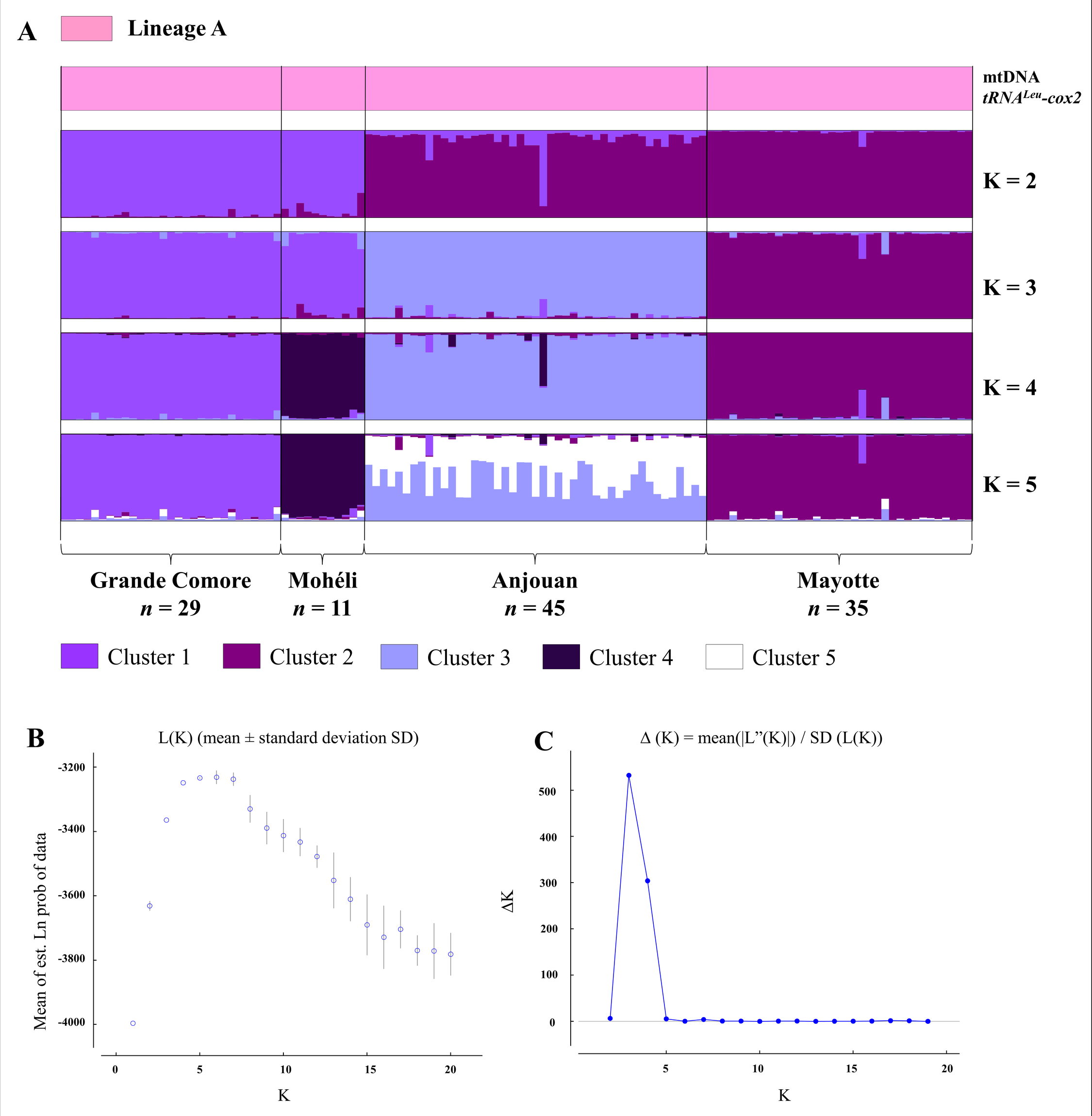

Supplement: S8 Fig — A) STRUCTURE bar plots from K = 2 to 5. All colonies had haplotypes from the COI-COII intergenic region characteristic of the African evolutionary lineage. B) Average likelihood of runs in STRUCTURE L(K) along with number of K clusters for Comoros Archipelago. C) ΔK, estimator of the optimal number of clusters (K) according to Evanno et al. (58). (TIF) [file pone.0189234.s008.tif]

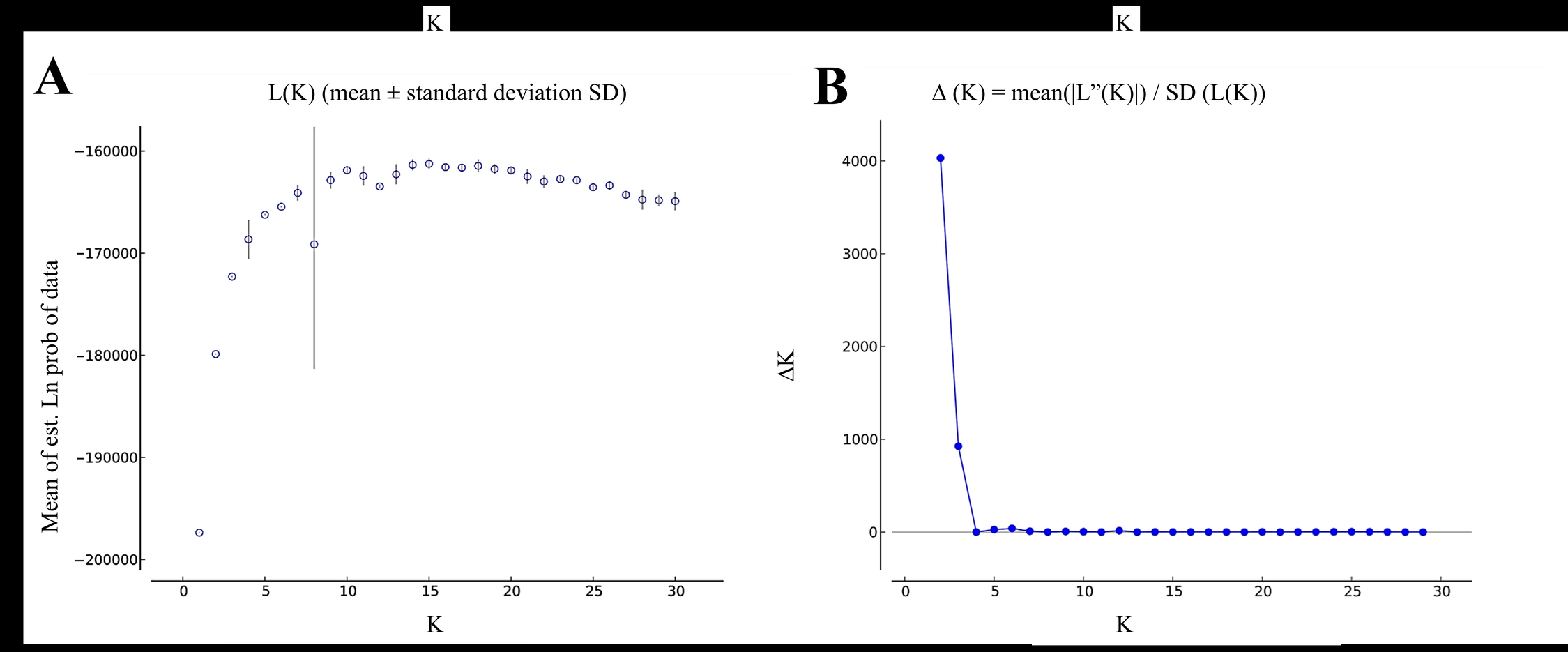

Supplement: S9 Fig — A) Average likelihood of runs in STRUCTURE L(K) along with number of K clusters for global STRUCTURE based on 4,388 honey bees Comoros Archipelago (Fig 6). B) ΔK, estimator of the optimal number of clusters (K) according to Evanno et al. (58). (TIF) [file pone.0189234.s009.tif]

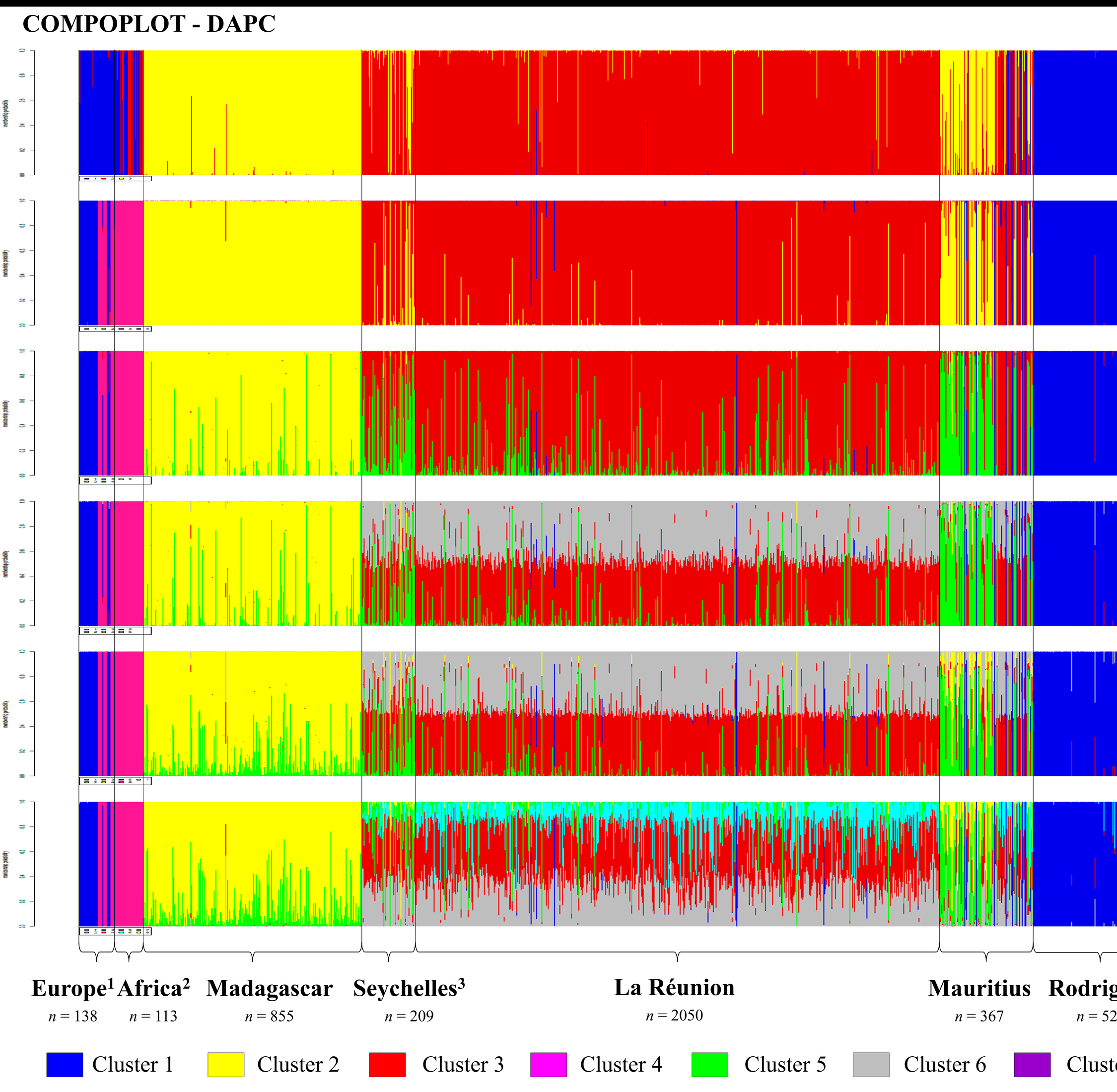

Supplement: S10 Fig — DAPC bar plots are presented for K = 3 to 8, based on 4,388 honey bees. (TIF) [file pone.0189234.s010.tif]

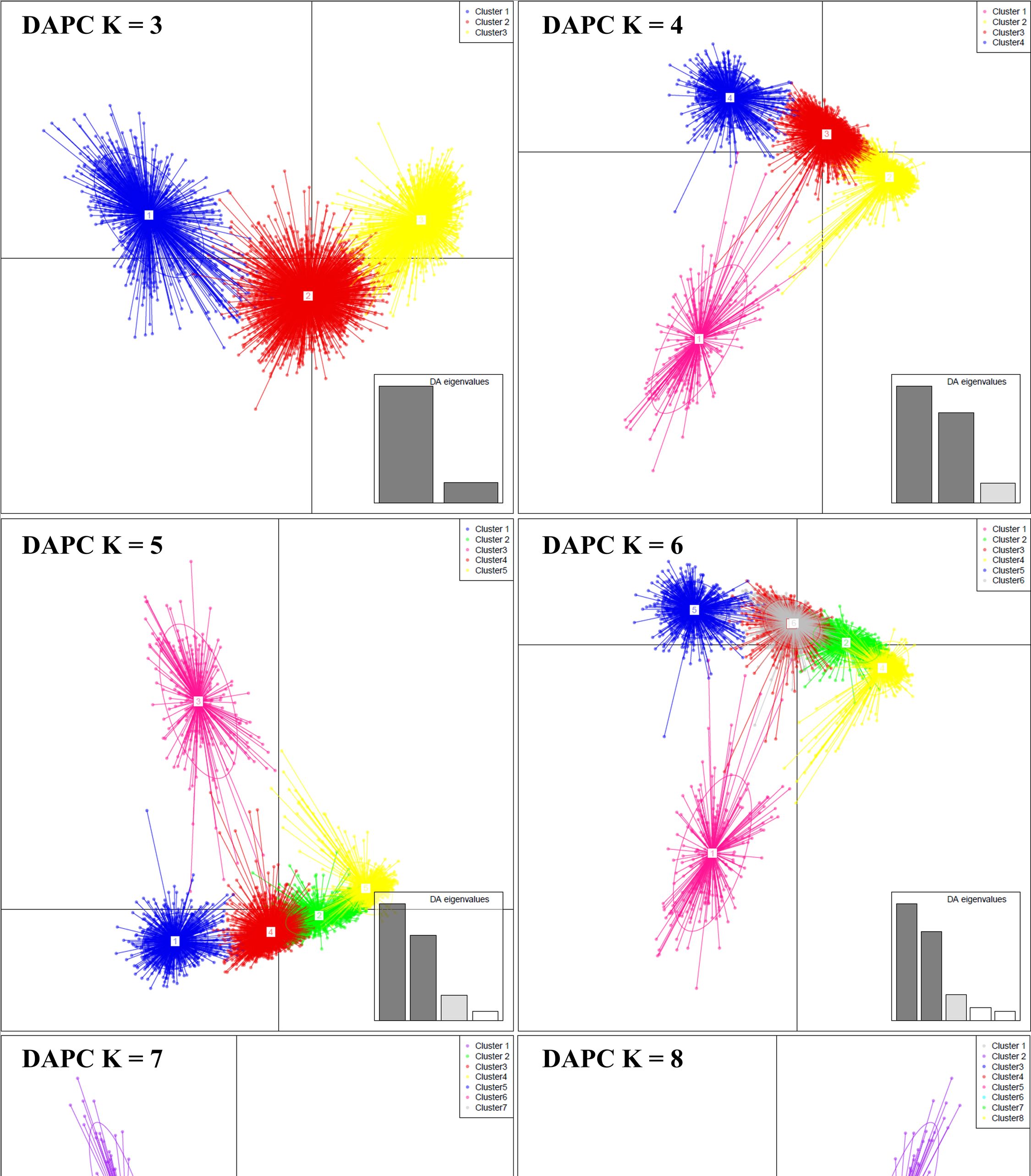

Supplement: S11 Fig — Colors of the different clusters correspond to the S10 Fig. (TIF) [file pone.0189234.s011.tif]
