# Supplementary material for: Genetic diversity and differentiation among insular honey bee populations in the southwest Indian Ocean likely reflect old geographical isolation and modern introductions
Source: PLoS One. 2017 Dec 27;12(12):e0189234. doi: 10.1371/journal.pone.0189234 (PMC5744932; doi:10.1371/journal.pone.0189234)
Supplement: S2 Table — N: number of colonies per site; NACOI-COII: number of individuals with missing COI-COII data, Nall: mean number of alleles; Hnb and Hobs: unbiased expected and observed heterozygosity, respectively; FIS (* significant at P < 0.05) and Anull: mean allele null frequency. (DOCX) [file pone.0189234.s013.docx]

**S2 Table**

|  |  | **P_0_Q** | | | **P_0_QQ** | | | | | **P_1_QQ** | | **P_1_QQQ** | **PQ** | | **PQQ** | | | **PQQQ** | **Q** |  |  |  |  |  |  |
| --- | --- | --- | --- | --- | --- | --- | --- | --- | --- | --- | --- | --- | --- | --- | --- | --- | --- | --- | --- | --- | --- | --- | --- | --- | --- |
|  | **N** | **A1** | **A49** | **Z7** | **A4** | **A6** | **A50** | **A51** | **Z2** | **A11** | **A14** | **A16** | **M3** | **M6** | **M4** | **M65** | **M7** | **M7'** | **C1/C2** | **NA_COI-COII_** | **N_all_** | **H_nb_** | **H_obs_** | **F_IS_** | **A_null_** |
| ***Madagascar*** |  |  |  |  |  |  |  |  |  |  |  |  |  |  |  |  |  |  |  |  |  |  |  |  |  |
| MDG77 | **12** | 12 |  |  |  |  |  |  |  |  |  |  |  |  |  |  |  |  |  |  | 2.86 | 0.399 | 0.393 | 0.018 | 0.017 |
| MDG78 | **26** | 26 |  |  |  |  |  |  |  |  |  |  |  |  |  |  |  |  |  |  | 4.29 | 0.466 | 0.448 | 0.041* | 0.031 |
| MDG79 | **1** | 1 |  |  |  |  |  |  |  |  |  |  |  |  |  |  |  |  |  |  | - | - | - | - | - |
| MDG80 | **12** | 12 |  |  |  |  |  |  |  |  |  |  |  |  |  |  |  |  |  |  | 3.79 | 0.463 | 0.488 | -0.057 | 0.014 |
| MDG81 | **56** | 56 |  |  |  |  |  |  |  |  |  |  |  |  |  |  |  |  |  |  | 4.93 | 0.435 | 0.434 | 0.004 | 0.009 |
| ***La Réunion*** |  |  |  |  |  |  |  |  |  |  |  |  |  |  |  |  |  |  |  |  |  |  |  |  |  |
| REU001 | **3** | 3 |  |  |  |  |  |  |  |  |  |  |  |  |  |  |  |  |  |  | - | - | - | - | - |
| REU002 | **14** | 14 |  |  |  |  |  |  |  |  |  |  |  |  |  |  |  |  |  |  | 5.36 | 0.679 | 0.679 | 0.001 | 0.032 |
| REU003 | **10** | 8 |  |  |  |  |  |  |  |  |  |  |  |  |  |  |  |  |  | 2 | 5.36 | 0.689 | 0.742 | -0.082 | 0.009 |
| REU004 | **1** | 1 |  |  |  |  |  |  |  |  |  |  |  |  |  |  |  |  |  |  | - | - | - | - | - |
| REU005 | **32** | 32 |  |  |  |  |  |  |  |  |  |  |  |  |  |  |  |  |  |  | 7.14 | 0.645 | 0.649 | -0.006 | 0.009 |
| REU006 | **1** | 1 |  |  |  |  |  |  |  |  |  |  |  |  |  |  |  |  |  |  | - | - | - | - | - |
| REU007 | **1** | 1 |  |  |  |  |  |  |  |  |  |  |  |  |  |  |  |  |  |  | - | - | - | - | - |
| REU008 | **1** | 1 |  |  |  |  |  |  |  |  |  |  |  |  |  |  |  |  |  |  | - | - | - | - | - |
| REU009 | **1** | 1 |  |  |  |  |  |  |  |  |  |  |  |  |  |  |  |  |  |  | - | - | - | - | - |
| REU010 | **1** | 1 |  |  |  |  |  |  |  |  |  |  |  |  |  |  |  |  |  |  | - | - | - | - | - |
| REU011 | **42** | 33 |  |  |  |  |  |  |  |  |  |  |  |  |  |  |  |  | 9 |  | 7.00 | 0.648 | 0.656 | -0.011 | 0.009 |
| REU012 | **2** | 2 |  |  |  |  |  |  |  |  |  |  |  |  |  |  |  |  |  |  | - | - | - | - | - |
| REU013 | **29** | 29 |  |  |  |  |  |  |  |  |  |  |  |  |  |  |  |  |  |  | 6.00 | 0.618 | 0.594 | 0.038 | 0.025 |
| REU014 | **38** | 38 |  |  |  |  |  |  |  |  |  |  |  |  |  |  |  |  |  |  | 7.21 | 0.655 | 0.618 | 0.058* | 0.022 |
| REU015 | **5** | 5 |  |  |  |  |  |  |  |  |  |  |  |  |  |  |  |  |  |  | 3.86 | 0.664 | 0.614 | 0.083 | 0.019 |
| REU016 | **9** | 9 |  |  |  |  |  |  |  |  |  |  |  |  |  |  |  |  |  |  | 4.71 | 0.694 | 0.699 | -0.007 | 0.010 |
| REU017 | **5** | 5 |  |  |  |  |  |  |  |  |  |  |  |  |  |  |  |  |  |  | 3.29 | 0.549 | 0.543 | 0.013 | 0.038 |
| REU018 | **1** | 1 |  |  |  |  |  |  |  |  |  |  |  |  |  |  |  |  |  |  | - | - | - | - | - |
| REU019 | **31** | 31 |  |  |  |  |  |  |  |  |  |  |  |  |  |  |  |  |  |  | 6.29 | 0.672 | 0.631 | 0.063 | 0.028 |
| REU020 | **30** | 30 |  |  |  |  |  |  |  |  |  |  |  |  |  |  |  |  |  |  | 7.07 | 0.676 | 0.653 | 0.036 | 0.020 |
| REU021 | **63** | 63 |  |  |  |  |  |  |  |  |  |  |  |  |  |  |  |  |  |  | 8.07 | 0.665 | 0.669 | -0.005* | 0.014 |
| REU022 | **4** | 4 |  |  |  |  |  |  |  |  |  |  |  |  |  |  |  |  |  |  | - | - | - | - | - |
| REU023 | **21** | 20 |  |  |  |  |  |  |  |  |  |  |  |  |  |  |  |  | 1 |  | 7.43 | 0.679 | 0.699 | -0.030 | 0.012 |
| REU024 | **8** | 8 |  |  |  |  |  |  |  |  |  |  |  |  |  |  |  |  |  |  | 4.79 | 0.644 | 0.622 | 0.036 | 0.010 |
| REU025 | **27** | 23 |  |  |  |  |  |  |  |  |  |  |  |  |  |  |  |  | 4 |  | 6.71 | 0.678 | 0.667 | 0.017 | 0.013 |
| REU026 | **4** | 4 |  |  |  |  |  |  |  |  |  |  |  |  |  |  |  |  |  |  | - | - | - | - | - |
| REU027 | **4** | 4 |  |  |  |  |  |  |  |  |  |  |  |  |  |  |  |  |  |  | - | - | - | - | - |
| REU028 | **1** | 1 |  |  |  |  |  |  |  |  |  |  |  |  |  |  |  |  |  |  | - | - | - | - | - |
| REU029 | **29** | 25 |  |  |  |  |  |  |  |  |  |  |  |  |  |  |  |  |  | 4 | 6.07 | 0.659 | 0.663 | -0.006 | 0.016 |
| REU030 | **20** | 20 |  |  |  |  |  |  |  |  |  |  |  |  |  |  |  |  |  |  | 5.36 | 0.634 | 0.668 | -0.055 | 0.006 |
| REU031 | **20** | 20 |  |  |  |  |  |  |  |  |  |  |  |  |  |  |  |  |  |  | 6.86 | 0.698 | 0.704 | -0.008 | 0.011 |
| REU032 | **99** | 96 |  |  |  |  |  |  |  |  |  |  |  |  | 1 |  |  |  | 2 |  | 9.5 | 0.667 | 0.682 | -0.023 | 0.006 |
| REU033 | **21** | 20 |  |  |  |  |  |  |  |  |  |  |  |  |  |  |  |  | 1 |  | 6.57 | 0.677 | 0.661 | 0.025* | 0.022 |
| REU034 | **34** | 28 |  |  |  |  |  |  |  |  |  |  |  |  |  |  |  |  | 4 | 2 | 7.71 | 0.694 | 0.674 | 0.028* | 0.022 |
| REU035 | **24** | 22 |  |  |  |  |  |  |  |  |  |  |  |  |  |  |  |  | 2 |  | 6.64 | 0.670 | 0.660 | 0.016* | 0.021 |
| REU036 | **23** | 23 |  |  |  |  |  |  |  |  |  |  |  |  |  |  |  |  |  |  | 6.93 | 0.682 | 0.723 | -0.062 | 0.005 |
| REU037 | **1** |  |  |  |  |  |  |  |  |  |  |  |  |  |  |  |  |  | 1 |  | - | - | - | - | - |
| REU038 | **5** | 4 |  |  |  |  |  |  |  |  |  |  |  |  |  |  |  |  | 1 |  | 3.43 | 0.624 | 0.569 | 0.096* | 0.033 |
| REU039 | **74** | 71 |  |  |  |  |  |  |  |  |  |  |  |  |  |  |  |  | 3 |  | 8.07 | 0.663 | 0.667 | -0.006 | 0.008 |
| REU040 | **7** | 5 |  |  |  |  |  |  |  |  |  |  |  |  | 2 |  |  |  |  |  | 4.50 | 0.656 | 0.655 | 0.002 | 0.002 |
| REU041 | **37** | 35 |  |  |  |  |  |  |  |  |  |  |  |  |  |  |  |  | 2 |  | 7.50 | 0.659 | 0.632 | 0.041* | 0.020 |
| REU042 | **55** | 55 |  |  |  |  |  |  |  |  |  |  |  |  |  |  |  |  |  |  | 8.07 | 0.690 | 0.708 | -0.026* | 0.004 |
| REU043 | **50** | 39 |  |  |  |  |  |  |  |  |  |  |  |  |  |  |  |  | 11 |  | 8.00 | 0.678 | 0.674 | 0.005 | 0.010 |
| REU044 | **3** | 3 |  |  |  |  |  |  |  |  |  |  |  |  |  |  |  |  |  |  | - | - | - | - | - |
| REU045 | **5** | 5 |  |  |  |  |  |  |  |  |  |  |  |  |  |  |  |  |  |  | 3.79 | 0.684 | 0.750 | -0.111* | 0.003 |
| REU046 | **25** | 20 |  |  |  |  |  |  |  |  |  |  |  |  | 1 |  |  |  | 4 |  | 6.93 | 0.692 | 0.734 | -0.062 | 0.007 |
| REU047 | **9** | 8 |  |  |  |  |  |  |  |  |  |  |  |  |  |  |  |  |  | 1 | 5.07 | 0.686 | 0.698 | -0.020 | 0.018 |
| REU048 | **6** | 6 |  |  |  |  |  |  |  |  |  |  |  |  |  |  |  |  |  |  | 4.14 | 0.672 | 0.700 | -0.046 | 0.009 |
| REU049 | **20** | 20 |  |  |  |  |  |  |  |  |  |  |  |  |  |  |  |  |  |  | 6.86 | 0.693 | 0.688 | 0.006 | 0.014 |
| REU050 | **1** | 1 |  |  |  |  |  |  |  |  |  |  |  |  |  |  |  |  |  |  | - | - | - | - | - |
| REU051 | **3** | 2 |  |  | 1 |  |  |  |  |  |  |  |  |  |  |  |  |  |  |  | - | - | - | - | - |
| REU052 | **18** | 16 |  |  |  |  |  |  |  |  |  |  |  |  | 1 |  |  |  | 1 |  | 6.00 | 0.682 | 0.680 | 0.004 | 0.024 |
| REU053 | **80** | 71 |  |  |  |  |  |  |  |  |  |  |  |  |  |  |  |  | 8 | 1 | 9.07 | 0.677 | 0.652 | 0.038* | 0.019 |
| REU054 | **2** | 2 |  |  |  |  |  |  |  |  |  |  |  |  |  |  |  |  |  |  | - | - | - | - | - |
| REU055 | **20** | 20 |  |  |  |  |  |  |  |  |  |  |  |  |  |  |  |  |  |  | 6.86 | 0.711 | 0.703 | 0.012 | 0.015 |
| REU056 | **41** | 38 |  |  | 2 |  |  |  |  |  |  |  |  |  |  |  |  |  | 1 |  | 8.29 | 0.682 | 0.673 | 0.014 | 0.009 |
| REU057 | **9** | 9 |  |  |  |  |  |  |  |  |  |  |  |  |  |  |  |  |  |  | 4.79 | 0.642 | 0.682 | -0.067 | 0.000 |
| REU058 | **3** | 3 |  |  |  |  |  |  |  |  |  |  |  |  |  |  |  |  |  |  | - | - | - | - | - |
| REU059 | **11** | 11 |  |  |  |  |  |  |  |  |  |  |  |  |  |  |  |  |  |  | 4.93 | 0.646 | 0.675 | -0.048 | 0.019 |
| REU060 | **13** | 11 |  |  |  |  |  |  |  |  |  |  |  |  |  |  |  |  | 1 | 1 | 6.07 | 0.662 | 0.648 | 0.022 | 0.011 |
| REU061 | **1** | 1 |  |  |  |  |  |  |  |  |  |  |  |  |  |  |  |  |  |  | - | - | - | - | - |
| REU062 | **2** | 2 |  |  |  |  |  |  |  |  |  |  |  |  |  |  |  |  |  |  | - | - | - | - | - |
| REU063 | **5** | 5 |  |  |  |  |  |  |  |  |  |  |  |  |  |  |  |  |  |  | 3.93 | 0.654 | 0.643 | 0.019 | 0.024 |
| REU064 | **6** | 5 |  |  |  |  |  |  |  |  |  |  |  |  |  |  |  |  |  | 1 | 4.36 | 0.687 | 0.625 | 0.098 | 0.037 |
| REU065 | **9** | 9 |  |  |  |  |  |  |  |  |  |  |  |  |  |  |  |  |  |  | 4.64 | 0.657 | 0.651 | 0.010 | 0.015 |
| REU066 | **4** | 4 |  |  |  |  |  |  |  |  |  |  |  |  |  |  |  |  |  |  | - | - | - | - | - |
| REU067 | **1** |  |  |  |  |  |  |  |  |  |  |  |  |  |  |  |  |  | 1 |  | - | - | - | - | - |
| REU068 | **3** | 2 |  |  |  |  |  |  |  |  |  |  |  |  |  |  |  |  |  | 1 | - | - | - | - | - |
| REU069 | **7** | 7 |  |  |  |  |  |  |  |  |  |  |  |  |  |  |  |  |  |  | 4.64 | 0.678 | 0.651 | 0.042 | 0.006 |
| REU070 | **24** | 19 |  |  |  |  |  |  |  |  |  |  |  |  |  |  |  |  |  | 5 | 7.50 | 0.705 | 0.691 | 0.020 | 0.022 |
| REU071 | **10** | 10 |  |  |  |  |  |  |  |  |  |  |  |  |  |  |  |  |  |  | 4.93 | 0.676 | 0.700 | -0.037 | 0.011 |
| REU072 | **21** | 19 |  |  |  |  |  |  |  |  |  |  |  |  |  |  |  |  |  | 2 | 6.57 | 0.708 | 0.739 | -0.044 | 0.003 |
| REU073 | **2** | 2 |  |  |  |  |  |  |  |  |  |  |  |  |  |  |  |  |  |  | - | - | - | - | - |
| REU074 | **2** | 2 |  |  |  |  |  |  |  |  |  |  |  |  |  |  |  |  |  |  | - | - | - | - | - |
| REU075 | **3** | 3 |  |  |  |  |  |  |  |  |  |  |  |  |  |  |  |  |  |  | - | - | - | - | - |
| REU076 | **21** | 14 |  |  |  |  |  |  |  |  |  |  |  |  |  |  |  |  | 2 | 5 | 7.21 | 0.701 | 0.735 | -0.049 | 0.009 |
| REU077 | **6** | 6 |  |  |  |  |  |  |  |  |  |  |  |  |  |  |  |  |  |  | 4.71 | 0.702 | 0.655 | 0.074 | 0.037 |
| REU078 | **1** | 1 |  |  |  |  |  |  |  |  |  |  |  |  |  |  |  |  |  |  | - | - | - | - | - |
| REU079 | **10** | 10 |  |  |  |  |  |  |  |  |  |  |  |  |  |  |  |  |  |  | 5.29 | 0.686 | 0.674 | 0.019 | 0.030 |
| REU080 | **20** | 18 |  |  |  |  |  |  |  |  |  |  |  |  |  |  |  |  | 1 | 1 | 7.00 | 0.695 | 0.683 | 0.019 | 0.022 |
| REU081 | **6** | 6 |  |  |  |  |  |  |  |  |  |  |  |  |  |  |  |  |  |  | 4.79 | 0.711 | 0.702 | 0.013 | 0.016 |
| REU082 | **20** | 20 |  |  |  |  |  |  |  |  |  |  |  |  |  |  |  |  |  |  | 6.86 | 0.684 | 0.638 | 0.069* | 0.031 |
| REU083 | **1** | 1 |  |  |  |  |  |  |  |  |  |  |  |  |  |  |  |  |  |  | - | - | - | - | - |
| REU084 | **1** | 1 |  |  |  |  |  |  |  |  |  |  |  |  |  |  |  |  |  |  | - | - | - | - | - |
| REU085 | **15** | 12 |  |  |  |  |  |  |  |  |  |  |  |  |  |  |  |  | 2 | 1 | 6.07 | 0.684 | 0.637 | 0.070* | 0.027 |
| REU086 | **40** | 40 |  |  |  |  |  |  |  |  |  |  |  |  |  |  |  |  |  |  | 8.14 | 0.682 | 0.644 | 0.056* | 0.024 |
| REU087 | **20** | 18 |  |  |  |  |  |  |  |  |  |  |  |  |  |  |  |  |  | 2 | 6.86 | 0.683 | 0.648 | 0.051* | 0.024 |
| REU088 | **27** | 24 |  |  |  |  |  |  |  |  |  |  |  |  |  |  |  |  | 1 | 2 | 7.29 | 0.697 | 0.685 | 0.017* | 0.018 |
| REU089 | **24** | 13 |  |  |  |  |  |  |  |  |  |  |  |  |  |  |  |  | 6 | 5 | 7.14 | 0.700 | 0.718 | -0.026 | 0.008 |
| REU090 | **20** | 16 |  |  | 2 |  |  |  |  |  |  |  |  |  |  |  |  |  |  | 2 | 6.21 | 0.682 | 0.679 | 0.004 | 0.013 |
| REU091 | **27** | 23 |  |  |  |  |  |  |  |  |  |  |  |  |  |  |  |  | 4 |  | 6.93 | 0.674 | 0.641 | 0.050* | 0.026 |
| REU092 | **20** | 17 |  |  |  |  |  |  |  |  |  |  |  |  |  |  |  |  |  | 3 | 6.50 | 0.687 | 0.697 | -0.016 | 0.014 |
| REU093 | **31** | 27 |  |  |  |  |  |  |  |  |  |  |  |  |  |  |  |  | 2 | 2 | 7.43 | 0.693 | 0.677 | 0.023 | 0.018 |
| REU094 | **15** | 15 |  |  |  |  |  |  |  |  |  |  |  |  |  |  |  |  |  |  | 5.36 | 0.656 | 0.650 | 0.009 | 0.017 |
| REU095 | **2** | 2 |  |  |  |  |  |  |  |  |  |  |  |  |  |  |  |  |  |  | - | - | - | - | - |
| REU096 | **11** | 7 |  |  |  |  |  |  |  |  |  |  |  |  |  |  |  |  | 4 |  | 5.43 | 0.687 | 0.675 | 0.017 | 0.022 |
| REU097 | **9** | 9 |  |  |  |  |  |  |  |  |  |  |  |  |  |  |  |  |  |  | 4.71 | 0.681 | 0.610 | 0.110 | 0.035 |
| REU098 | **1** | 1 |  |  |  |  |  |  |  |  |  |  |  |  |  |  |  |  |  |  | - | - | - | - | - |
| REU099 | **22** | 13 |  |  |  |  |  |  |  |  |  |  |  |  |  |  |  |  | 1 | 8 | 6.79 | 0.671 | 0.636 | 0.053 | 0.021 |
| REU100 | **50** | 50 |  |  |  |  |  |  |  |  |  |  |  |  |  |  |  |  |  |  | 8.71 | 0.688 | 0.695 | -0.010 | 0.006 |
| REU101 | **2** | 2 |  |  |  |  |  |  |  |  |  |  |  |  |  |  |  |  |  |  | - | - | - | - | - |
| REU102 | **68** | 66 |  |  |  |  |  |  |  |  |  |  |  |  |  |  |  |  | 2 |  | 8.64 | 0.659 | 0.646 | 0.019 | 0.011 |
| REU103 | **6** | 6 |  |  |  |  |  |  |  |  |  |  |  |  |  |  |  |  |  |  | 4.07 | 0.637 | 0.662 | -0.042 | 0.010 |
| REU104 | **5** | 5 |  |  |  |  |  |  |  |  |  |  |  |  |  |  |  |  |  |  | 3.79 | 0.628 | 0.536 | 0.160* | 0.030 |
| REU105 | **1** | 1 |  |  |  |  |  |  |  |  |  |  |  |  |  |  |  |  |  |  | - | - | - | - | - |
| REU106 | **1** | 1 |  |  |  |  |  |  |  |  |  |  |  |  |  |  |  |  |  |  | - | - | - | - | - |
| REU107 | **2** | 2 |  |  |  |  |  |  |  |  |  |  |  |  |  |  |  |  |  |  | - | - | - | - | - |
| REU108 | **22** | 9 |  |  |  |  |  |  |  |  |  |  |  |  |  |  |  |  |  | 13 | 6.64 | 0.692 | 0.659 | 0.049* | 0.030 |
| REU109 | **29** | 29 |  |  |  |  |  |  |  |  |  |  |  |  |  |  |  |  |  |  | 7.07 | 0.681 | 0.643 | 0.056* | 0.022 |
| REU110 | **13** | 13 |  |  |  |  |  |  |  |  |  |  |  |  |  |  |  |  |  |  | 5.36 | 0.653 | 0.673 | -0.031 | 0.010 |
| REU111 | **2** | 2 |  |  |  |  |  |  |  |  |  |  |  |  |  |  |  |  |  |  | - | - | - | - | - |
| REU112 | **16** | 16 |  |  |  |  |  |  |  |  |  |  |  |  |  |  |  |  |  |  | 5.50 | 0.634 | 0.612 | 0.036 | 0.020 |
| REU113 | **10** | 10 |  |  |  |  |  |  |  |  |  |  |  |  |  |  |  |  |  |  | 4.50 | 0.615 | 0.589 | 0.046* | 0.033 |
| REU114 | **2** | 2 |  |  |  |  |  |  |  |  |  |  |  |  |  |  |  |  |  |  | - | - | - | - | - |
| REU115 | **5** | 5 |  |  |  |  |  |  |  |  |  |  |  |  |  |  |  |  |  |  | 3.57 | 0.635 | 0.643 | -0.014 | 0.022 |
| REU116 | **17** | 15 |  |  |  |  |  |  |  |  |  |  |  |  |  |  |  |  | 1 | 1 | 6.14 | 0.663 | 0.662 | 0.001 | 0.020 |
| REU117 | **1** | 1 |  |  |  |  |  |  |  |  |  |  |  |  |  |  |  |  |  |  | - | - | - | - | - |
| REU118 | **10** | 10 |  |  |  |  |  |  |  |  |  |  |  |  |  |  |  |  |  |  | 4.71 | 0.638 | 0.637 | 0.003 | 0.016 |
| REU119 | **7** | 7 |  |  |  |  |  |  |  |  |  |  |  |  |  |  |  |  |  |  | 4.00 | 0.641 | 0.643 | -0.003 | 0.019 |
| REU120 | **14** | 13 |  |  |  |  |  |  |  |  |  |  |  |  |  |  |  |  |  | 1 | 5.86 | 0.675 | 0.671 | 0.007 | 0.010 |
| REU121 | **1** | 1 |  |  |  |  |  |  |  |  |  |  |  |  |  |  |  |  |  |  | - | - | - | - | - |
| REU122 | **7** | 7 |  |  |  |  |  |  |  |  |  |  |  |  |  |  |  |  |  |  | 3.71 | 0.650 | 0.668 | -0.031 | 0.022 |
| REU123 | **3** | 2 |  |  |  |  |  |  |  |  |  |  |  |  |  |  |  |  |  | 1 | - | - | - | - | - |
| REU124 | **40** | 40 |  |  |  |  |  |  |  |  |  |  |  |  |  |  |  |  |  |  | 8.00 | 0.693 | 0.672 | 0.030* | 0.018 |
| REU125 | **5** | 5 |  |  |  |  |  |  |  |  |  |  |  |  |  |  |  |  |  |  | 3.93 | 0.689 | 0.643 | 0.075 | 0.032 |
| REU126 | **60** | 58 |  |  |  |  |  |  |  |  |  |  |  |  |  |  |  |  |  | 2 | 8.29 | 0.684 | 0.662 | 0.032* | 0.022 |
| REU127 | **24** | 22 |  |  |  |  |  |  |  |  |  |  |  |  |  |  |  |  | 2 |  | 6.36 | 0.695 | 0.728 | -0.049 | 0.002 |
| ***Mauritius*** |  |  |  |  |  |  |  |  |  |  |  |  |  |  |  |  |  |  |  |  |  |  |  |  |  |
| MUS01 | **1** |  |  |  |  |  |  |  |  |  |  |  |  |  |  |  |  |  | 1 |  | - | - | - | - | - |
| MUS02 | **20** | 19 | 1 |  |  |  |  |  |  |  |  |  |  |  |  |  |  |  |  |  | 3.79 | 0.391 | 0.414 | -0.060 | 0.008 |
| MUS03 | **1** |  |  |  |  |  |  |  |  |  |  |  |  |  |  |  |  |  | 1 |  | - | - | - | - | - |
| MUS04 | **16** | 15 | 1 |  |  |  |  |  |  |  |  |  |  |  |  |  |  |  |  |  | 4.36 | 0.441 | 0.420 | 0.050 | 0.016 |
| MUS05 | **3** | 1 |  |  |  |  |  |  |  |  |  |  |  |  |  |  |  |  | 2 |  | - | - | - | - | - |
| MUS06 | **10** | 10 |  |  |  |  |  |  |  |  |  |  |  |  |  |  |  |  |  |  | 2.71 | 0.358 | 0.357 | 0.003 | 0.011 |
| MUS07 | **13** | 12 |  |  |  |  |  |  |  |  |  |  |  |  |  |  |  |  | 1 |  | 4.07 | 0.450 | 0.445 | 0.012 | 0.009 |
| MUS08 | **22** | 18 |  |  |  |  |  |  |  |  |  |  |  |  |  |  | 2 |  | 2 |  | 5.79 | 0.538 | 0.513 | 0.048* | 0.033 |
| MUS09 | **29** | 11 |  |  |  |  |  |  |  |  |  |  |  |  |  |  | 2 |  | 16 |  | 6.64 | 0.632 | 0.613 | 0.029 | 0.020 |
| MUS10 | **1** | 1 |  |  |  |  |  |  |  |  |  |  |  |  |  |  |  |  |  |  | - | - | - | - | - |
| MUS11 | **2** | 2 |  |  |  |  |  |  |  |  |  |  |  |  |  |  |  |  |  |  | - | - | - | - | - |
| MUS12 | **23** | 22 |  |  |  |  |  |  |  |  |  |  |  |  |  |  |  |  | 1 |  | 4.86 | 0.406 | 0.385 | 0.053 | 0.009 |
| MUS13 | **4** | 1 |  |  |  |  |  |  |  |  |  |  |  |  |  |  |  |  | 3 |  | - | - | - | - | - |
| MUS14 | **1** | 1 |  |  |  |  |  |  |  |  |  |  |  |  |  |  |  |  |  |  | - | - | - | - | - |
| MUS15 | **8** | 4 |  |  |  |  |  |  |  |  |  |  |  |  |  |  |  |  | 4 |  | 2.79 | 0.396 | 0.402 | -0.016 | 0.019 |
| MUS16 | **14** | 14 |  |  |  |  |  |  |  |  |  |  |  |  |  |  |  |  |  |  | 4.00 | 0.413 | 0.408 | 0.012 | 0.010 |
| MUS17 | **14** | 12 |  |  | 1 |  |  |  |  |  |  |  |  |  |  |  |  |  | 1 |  | 5.00 | 0.525 | 0.500 | 0.050 | 0.028 |
| MUS18 | **28** | 27 |  |  |  |  |  |  |  |  |  |  |  |  |  |  |  |  | 1 |  | 5.79 | 0.466 | 0.466 | -0.001 | 0.011 |
| MUS19 | **19** | 4 |  |  |  |  |  |  |  |  |  |  |  |  |  |  |  |  | 15 |  | 5.50 | 0.612 | 0.623 | -0.019 | 0.019 |
| MUS20 | **93** |  |  |  |  |  |  |  |  |  |  |  |  |  |  |  |  |  | 93 |  | 8.79 | 0.691 | 0.719 | -0.040 | 0.008 |
| MUS21 | **20** | 1 |  |  |  |  |  |  |  |  |  |  |  |  |  |  |  |  | 19 |  | 7.00 | 0.680 | 0.754 | -0.111* | 0.002 |
| MUS22 | **1** | 1 |  |  |  |  |  |  |  |  |  |  |  |  |  |  |  |  |  |  | - | - | - | - | - |
| MUS23 | **1** | 1 |  |  |  |  |  |  |  |  |  |  |  |  |  |  |  |  |  |  | - | - | - | - | - |
| MUS24 | **2** | 2 |  |  |  |  |  |  |  |  |  |  |  |  |  |  |  |  |  |  | - | - | - | - | - |
| MUS25 | **4** | 4 |  |  |  |  |  |  |  |  |  |  |  |  |  |  |  |  |  |  | - | - | - | - | - |
| MUS26 | **5** | 3 |  |  |  |  |  |  |  |  |  |  |  |  |  |  |  |  | 2 |  | 3.07 | 0.521 | 0.492 | 0.059 | 0.031 |
| MUS27 | **1** | 1 |  |  |  |  |  |  |  |  |  |  |  |  |  |  |  |  |  |  | - | - | - | - | - |
| MUS28 | **4** | 4 |  |  |  |  |  |  |  |  |  |  |  |  |  |  |  |  |  |  | - | - | - | - | - |
| MUS29 | **1** | 1 |  |  |  |  |  |  |  |  |  |  |  |  |  |  |  |  |  |  | - | - | - | - | - |
| MUS30 | **2** | 1 |  |  |  |  |  |  |  |  |  |  |  |  |  |  |  |  | 1 |  | - | - | - | - | - |
| MUS31 | **4** | 3 |  |  |  |  |  |  |  |  |  |  |  |  |  |  |  |  | 1 |  | - | - | - | - | - |
| ***Mahé (2015)*** |  |  |  |  |  |  |  |  |  |  |  |  |  |  |  |  |  |  |  |  |  |  |  |  |  |
| SYC33 | **2** | 2 |  |  |  |  |  |  |  |  |  |  |  |  |  |  |  |  |  |  | - | - | - | - | - |
| SYC34 | **3** | 3 |  |  |  |  |  |  |  |  |  |  |  |  |  |  |  |  |  |  | - | - | - | - | - |
| SYC35 | **1** | 1 |  |  |  |  |  |  |  |  |  |  |  |  |  |  |  |  |  |  | - | - | - | - | - |
| SYC36 | **2** | 2 |  |  |  |  |  |  |  |  |  |  |  |  |  |  |  |  |  |  | - | - | - | - | - |
| SYC37 | **2** | 2 |  |  |  |  |  |  |  |  |  |  |  |  |  |  |  |  |  |  | - | - | - | - | - |
| ***Praslin (2015)*** |  |  |  |  |  |  |  |  |  |  |  |  |  |  |  |  |  |  |  |  |  |  |  |  |  |
| SYC38 | **2** | 2 |  |  |  |  |  |  |  |  |  |  |  |  |  |  |  |  |  |  | - | - | - | - | - |
| SYC39 | **3** | 3 |  |  |  |  |  |  |  |  |  |  |  |  |  |  |  |  |  |  | - | - | - | - | - |
| SYC40 | **2** | 1 |  |  |  |  |  |  | 1 |  |  |  |  |  |  |  |  |  |  |  | - | - | - | - | - |
| SYC41 | **1** |  |  |  |  |  |  |  | 1 |  |  |  |  |  |  |  |  |  |  |  | - | - | - | - | - |
| ***La Digue (2015)*** |  |  |  |  |  |  |  |  |  |  |  |  |  |  |  |  |  |  |  |  |  |  |  |  |  |
| SYC42 | **3** | 3 |  |  |  |  |  |  |  |  |  |  |  |  |  |  |  |  |  |  | - | - | - | - | - |
| SYC43 | **1** | 1 |  |  |  |  |  |  |  |  |  |  |  |  |  |  |  |  |  |  | - | - | - | - | - |
| SYC44 | **2** | 2 |  |  |  |  |  |  |  |  |  |  |  |  |  |  |  |  |  |  | - | - | - | - | - |
| ***Grande Comore*** |  |  |  |  |  |  |  |  |  |  |  |  |  |  |  |  |  |  |  |  |  |  |  |  |  |
| GCO01 | **5** | 5 |  |  |  |  |  |  |  |  |  |  |  |  |  |  |  |  |  |  | 2.57 | 0.433 | 0.500 | -0.176* | 0.000 |
| GCO02 | **2** | 2 |  |  |  |  |  |  |  |  |  |  |  |  |  |  |  |  |  |  | - | - | - | - | - |
| GCO03 | **3** | 3 |  |  |  |  |  |  |  |  |  |  |  |  |  |  |  |  |  |  | - | - | - | - | - |
| GCO04 | **3** | 3 |  |  |  |  |  |  |  |  |  |  |  |  |  |  |  |  |  |  | - | - | - | - | - |
| GCO05 | **4** | 4 |  |  |  |  |  |  |  |  |  |  |  |  |  |  |  |  |  |  | - | - | - | - | - |
| GCO06 | **2** | 2 |  |  |  |  |  |  |  |  |  |  |  |  |  |  |  |  |  |  | - | - | - | - | - |
| GCO07 | **2** | 2 |  |  |  |  |  |  |  |  |  |  |  |  |  |  |  |  |  |  | - | - | - | - | - |
| GCO08 | **2** | 2 |  |  |  |  |  |  |  |  |  |  |  |  |  |  |  |  |  |  | - | - | - | - | - |
| GCO09 | **4** | 4 |  |  |  |  |  |  |  |  |  |  |  |  |  |  |  |  |  |  | - | - | - | - | - |
| GCO10 | **2** | 2 |  |  |  |  |  |  |  |  |  |  |  |  |  |  |  |  |  |  | - | - | - | - | - |
| ***Moheli*** |  |  |  |  |  |  |  |  |  |  |  |  |  |  |  |  |  |  |  |  |  |  |  |  |  |
| MOH01 | **6** | 6 |  |  |  |  |  |  |  |  |  |  |  |  |  |  |  |  |  |  | 3.33 | 0.592 | 0.359 | 0.440* | 0.207 |
| MOH02 | **2** | 2 |  |  |  |  |  |  |  |  |  |  |  |  |  |  |  |  |  |  | - | - | - | - | - |
| MOH03 | **3** | 3 |  |  |  |  |  |  |  |  |  |  |  |  |  |  |  |  |  |  | - | - | - | - | - |
| ***Anjouan*** |  |  |  |  |  |  |  |  |  |  |  |  |  |  |  |  |  |  |  |  |  |  |  |  |  |
| ANJ01 | **4** | 4 |  |  |  |  |  |  |  |  |  |  |  |  |  |  |  |  |  |  | - | - | - | - | - |
| ANJ02 | **4** | 4 |  |  |  |  |  |  |  |  |  |  |  |  |  |  |  |  |  |  | - | - | - | - | - |
| ANJ03 | **4** | 4 |  |  |  |  |  |  |  |  |  |  |  |  |  |  |  |  |  |  | - | - | - | - | - |
| ANJ04 | **4** | 4 |  |  |  |  |  |  |  |  |  |  |  |  |  |  |  |  |  |  | - | - | - | - | - |
| ANJ05 | **4** | 4 |  |  |  |  |  |  |  |  |  |  |  |  |  |  |  |  |  |  | - | - | - | - | - |
| ANJ06 | **4** | 4 |  |  |  |  |  |  |  |  |  |  |  |  |  |  |  |  |  |  | - | - | - | - | - |
| ANJ07 | **4** | 4 |  |  |  |  |  |  |  |  |  |  |  |  |  |  |  |  |  |  | - | - | - | - | - |
| ANJ08 | **3** | 3 |  |  |  |  |  |  |  |  |  |  |  |  |  |  |  |  |  |  | - | - | - | - | - |
| ANJ09 | **4** | 4 |  |  |  |  |  |  |  |  |  |  |  |  |  |  |  |  |  |  | - | - | - | - | - |
| ANJ10 | **2** | 2 |  |  |  |  |  |  |  |  |  |  |  |  |  |  |  |  |  |  | - | - | - | - | - |
| ANJ11 | **4** | 4 |  |  |  |  |  |  |  |  |  |  |  |  |  |  |  |  |  |  | - | - | - | - | - |
| ANJ12 | **1** | 1 |  |  |  |  |  |  |  |  |  |  |  |  |  |  |  |  |  |  | - | - | - | - | - |
| ANJ13 | **1** | 1 |  |  |  |  |  |  |  |  |  |  |  |  |  |  |  |  |  |  | - | - | - | - | - |
| ANJ14 | **1** | 1 |  |  |  |  |  |  |  |  |  |  |  |  |  |  |  |  |  |  | - | - | - | - | - |
| ANJ15 | **1** | 1 |  |  |  |  |  |  |  |  |  |  |  |  |  |  |  |  |  |  | - | - | - | - | - |
| ***Mayotte*** |  |  |  |  |  |  |  |  |  |  |  |  |  |  |  |  |  |  |  |  |  |  |  |  |  |
| MYT01 | **2** | 2 |  |  |  |  |  |  |  |  |  |  |  |  |  |  |  |  |  |  | - | - | - | - | - |
| MYT02 | **2** | 2 |  |  |  |  |  |  |  |  |  |  |  |  |  |  |  |  |  |  | - | - | - | - | - |
| MYT03 | **2** | 2 |  |  |  |  |  |  |  |  |  |  |  |  |  |  |  |  |  |  | - | - | - | - | - |
| MYT04 | **1** | 1 |  |  |  |  |  |  |  |  |  |  |  |  |  |  |  |  |  |  | - | - | - | - | - |
| MYT05 | **2** | 2 |  |  |  |  |  |  |  |  |  |  |  |  |  |  |  |  |  |  | - | - | - | - | - |
| MYT06 | **1** | 1 |  |  |  |  |  |  |  |  |  |  |  |  |  |  |  |  |  |  | - | - | - | - | - |
| MYT07 | **2** | 2 |  |  |  |  |  |  |  |  |  |  |  |  |  |  |  |  |  |  | - | - | - | - | - |
| MYT08 | **2** | 2 |  |  |  |  |  |  |  |  |  |  |  |  |  |  |  |  |  |  | - | - | - | - | - |
| MYT09 | **3** | 3 |  |  |  |  |  |  |  |  |  |  |  |  |  |  |  |  |  |  | - | - | - | - | - |
| MYT10 | **2** | 2 |  |  |  |  |  |  |  |  |  |  |  |  |  |  |  |  |  |  | - | - | - | - | - |
| MYT11 | **1** | 1 |  |  |  |  |  |  |  |  |  |  |  |  |  |  |  |  |  |  | - | - | - | - | - |
| MYT12 | **2** | 2 |  |  |  |  |  |  |  |  |  |  |  |  |  |  |  |  |  |  | - | - | - | - | - |
| MYT13 | **2** | 2 |  |  |  |  |  |  |  |  |  |  |  |  |  |  |  |  |  |  | - | - | - | - | - |
| MYT14 | **2** | 2 |  |  |  |  |  |  |  |  |  |  |  |  |  |  |  |  |  |  | - | - | - | - | - |
| MYT15 | **2** | 2 |  |  |  |  |  |  |  |  |  |  |  |  |  |  |  |  |  |  | - | - | - | - | - |
| MYT16 | **7** | 7 |  |  |  |  |  |  |  |  |  |  |  |  |  |  |  |  |  |  | 2.78 | 0.464 | 0.455 | 0.020 | 0.058 |
| ***Africa*** |  |  |  |  |  |  |  |  |  |  |  |  |  |  |  |  |  |  |  |  |  |  |  |  |  |
| EGY01 | **2** |  |  | 2 |  |  |  |  |  |  |  |  |  |  |  |  |  |  |  |  | - | - | - | - | - |
| SEN01 | **2** |  |  |  | 2 |  |  |  |  |  |  |  |  |  |  |  |  |  |  |  | - | - | - | - | - |
| STP01 | **12** |  |  |  | 10 |  | 1 |  |  |  |  |  |  |  |  |  |  |  |  | 1 | 4.79 | 0.653 | 0.693 | -0.063 | 0.014 |
| TCD01 | **1** |  |  |  |  | 1 |  |  |  |  |  |  |  |  |  |  |  |  |  |  | - | - | - | - | - |
| TCD02 | **3** |  |  |  | 2 |  |  |  |  |  |  |  |  |  |  |  |  |  |  | 1 | - | - | - | - | - |
| CAF01 | **3** | 2 |  |  | 1 |  |  |  |  |  |  |  |  |  |  |  |  |  |  |  | - | - | - | - | - |
| CAF02 | **2** | 1 |  |  | 1 |  |  |  |  |  |  |  |  |  |  |  |  |  |  |  | - | - | - | - | - |
| CAF03 | **1** |  |  |  | 1 |  |  |  |  |  |  |  |  |  |  |  |  |  |  |  | - | - | - | - | - |
| CAF04 | **3** | 2 |  |  | 1 |  |  |  |  |  |  |  |  |  |  |  |  |  |  |  | - | - | - | - | - |
| CAF05 | **2** | 1 |  |  | 1 |  |  |  |  |  |  |  |  |  |  |  |  |  |  |  | - | - | - | - | - |
| CMR01 | **6** | 3 |  |  | 2 |  |  |  |  |  |  |  |  |  |  |  |  |  |  | 1 |  |  |  |  |  |
| GAB01 | **1** | 1 |  |  |  |  |  |  |  |  |  |  |  |  |  |  |  |  |  |  | - | - | - | - | - |
| GAB02 | **2** | 1 |  |  | 1 |  |  |  |  |  |  |  |  |  |  |  |  |  |  |  | - | - | - | - | - |
| UGA01 | **1** |  |  |  |  |  |  | 1 |  |  |  |  |  |  |  |  |  |  |  |  | - | - | - | - | - |
| MWI01 | **3** |  |  |  |  |  |  |  |  |  |  |  |  |  |  |  |  |  |  | 3 | - | - | - | - | - |
| MWI02 | **1** | 1 |  |  |  |  |  |  |  |  |  |  |  |  |  |  |  |  |  |  | - | - | - | - | - |
| MWI03 | **2** | 2 |  |  |  |  |  |  |  |  |  |  |  |  |  |  |  |  |  |  | - | - | - | - | - |
| MWI04 | **2** | 2 |  |  |  |  |  |  |  |  |  |  |  |  |  |  |  |  |  |  | - | - | - | - | - |
| MOZ01 | **2** | 2 |  |  |  |  |  |  |  |  |  |  |  |  |  |  |  |  |  |  |  |  |  |  |  |
| TZA01 | **10** | 9 |  |  | 1 |  |  |  |  |  |  |  |  |  |  |  |  |  |  |  | 8.35 | 0.860 | 0.777 | 0.100* | 0.031 |
| TZA02 | **2** |  |  |  | 2 |  |  |  |  |  |  |  |  |  |  |  |  |  |  |  |  |  |  |  |  |
| TZA03 | **2** | 1 |  |  | 1 |  |  |  |  |  |  |  |  |  |  |  |  |  |  |  |  |  |  |  |  |
| ZAN01 | **3** | 3 |  |  |  |  |  |  |  |  |  |  |  |  |  |  |  |  |  |  |  |  |  |  |  |
| ZWE01 | **5** |  |  |  | 5 |  |  |  |  |  |  |  |  |  |  |  |  |  |  |  | 6.07 | 0.856 | 0.800 | 0.072 | 0.022 |
| ZWE02 | **9** |  |  |  | 9 |  |  |  |  |  |  |  |  |  |  |  |  |  |  |  | 8.43 | 0.870 | 0.831 | 0.047 | 0.017 |
| ZAF01 | **25** | 1 |  |  | 21 |  | 1 |  |  |  |  |  |  |  |  |  |  |  |  | 2 | 12.43 | 0.862 | 0.832 | 0.036 | 0.014 |
| ZAF02 | **2** |  |  |  | 1 |  |  |  |  |  |  |  |  |  |  |  |  |  |  | 1 | - | - | - | - | - |
| ZAF03 | **4** | 1 |  |  | 3 |  |  |  |  |  |  |  |  |  |  |  |  |  |  |  | - | - | - | - | - |
| ***Europe*** |  |  |  |  |  |  |  |  |  |  |  |  |  |  |  |  |  |  |  |  |  |  |  |  |  |
| CHE01 | **3** |  |  |  |  |  |  |  |  |  |  |  |  |  |  |  |  |  | 2 | 1 | - | - | - | - | - |
| DEU01 | **6** |  |  |  |  |  |  |  |  |  |  |  |  |  |  |  |  |  | 6 |  | 3.64 | 0.579 | 0.548 | 0.059 | 0.026 |
| DEU02 | **3** |  |  |  |  |  |  |  |  |  |  |  |  |  |  |  |  |  | 3 |  | - | - | - | - | - |
| ITA01 | **27** |  |  |  |  |  |  |  |  |  |  |  |  |  |  |  | 15 | 1 | 11 |  | 6.21 | 0.479 | 0.508 | -0.062* | 0.000 |
| ITA02 | **5** |  |  |  |  |  |  |  |  |  |  |  | 3 |  |  |  | 1 |  | 1 |  | 3.29 | 0.465 | 0.457 | 0.019 | 0.014 |
| ITA03 | **7** |  |  |  |  |  |  |  |  |  |  |  | 2 |  |  |  | 2 |  | 3 |  | 3.93 | 0.506 | 0.520 | -0.030 | 0.016 |
| ITA04 | **11** |  |  |  |  |  |  |  |  |  |  |  | 1 |  |  |  |  |  | 10 |  | 3.93 | 0.440 | 0.390 | 0.120* | 0.034 |
| ITA05 | **1** |  |  |  |  |  |  |  |  |  |  |  |  |  |  |  |  |  | 1 |  | - | - | - | - | - |
| ITA06 | **2** |  |  |  |  |  |  |  |  |  |  |  |  |  |  |  |  |  | 2 |  | - | - | - | - | - |
| ITA07 | **2** |  |  |  |  |  |  |  |  |  |  |  |  |  |  |  |  |  | 2 |  | - | - | - | - | - |
| ITA08 | **1** |  |  |  |  |  |  |  |  |  |  |  |  |  |  |  | 1 |  |  |  | - | - | - | - | - |
| GRC01 | **6** |  |  |  |  |  |  |  |  |  |  |  |  |  |  |  |  |  | 6 |  | 3.21 | 0.462 | 0.452 | 0.023 | 0.027 |
| FRA01 | **15** |  |  |  |  |  |  |  |  |  |  |  |  |  | 10 | 3 |  |  | 2 |  | 5.43 | 0.510 | 0.480 | 0.062 | 0.023 |
| FRA02 | **20** |  |  |  |  |  |  |  |  |  |  |  |  | 6 | 10 |  |  | 4 |  |  | 5.86 | 0.607 | 0.589 | 0.031* | 0.028 |
| FRA03 | **13** |  |  |  |  |  |  |  |  |  |  |  |  |  |  |  |  |  | 13 |  | 6.43 | 0.704 | 0.752 | -0.072 | 0.017 |
| ESP01 | **3** |  |  |  |  |  |  |  |  | 2 |  |  |  |  |  |  |  | 1 |  |  | - | - | - | - | - |
| PRT01 | **1** |  |  |  |  |  |  |  |  |  |  | 1 |  |  |  |  |  |  |  |  | - | - | - | - | - |
| PRT02 | **1** |  |  |  |  |  |  |  |  |  |  | 1 |  |  |  |  |  |  |  |  | - | - | - | - | - |
| PRT03 | **1** |  |  |  |  |  |  |  |  | 1 |  |  |  |  |  |  |  |  |  |  | - | - | - | - | - |
| PRT04 | **1** |  |  |  |  |  |  |  |  | 1 |  |  |  |  |  |  |  |  |  |  | - | - | - | - | - |
| PRT05 | **1** |  |  |  |  |  |  |  |  |  | 1 |  |  |  |  |  |  |  |  |  | - | - | - | - | - |
| PRT06 | **8** | 2 |  |  |  |  |  |  |  | 5 |  | 1 |  |  |  |  |  |  |  |  | 3.64 | 0.445 | 0.500 | -0.133* | 0.000 |
| ***Tahiti*** |  |  |  |  |  |  |  |  |  |  |  |  |  |  |  |  |  |  |  |  |  |  |  |  |  |
| TAH01 | **12** |  |  |  |  |  |  |  |  |  |  |  |  |  |  |  | 7 |  | 4 | 1 | 3.85 | 0.382 | 0.330 | 0.140* | 0.023 |
