# Supplementary material for: Genetic diversity and differentiation among insular honey bee populations in the southwest Indian Ocean likely reflect old geographical isolation and modern introductions
Source: PLoS One. 2017 Dec 27;12(12):e0189234. doi: 10.1371/journal.pone.0189234 (PMC5744932; doi:10.1371/journal.pone.0189234)
Supplement: S3 Table — After Bonferroni corrections, permutations tests were only significant among French sites (in bold P < 0.000549). Colors as in Table 3. (DOCX) [file pone.0189234.s014.docx]

**S3 Table**

|  | ZWE02 | FRA02 | FRA03 | ITA02 | ITA03 | ITA04 |
| --- | --- | --- | --- | --- | --- | --- |
| ZWE01 | 0.001 |  |  |  |  |  |
| FRA01 |  | **0.033** | **0.265** |  |  |  |
| FRA02 |  |  | **0.218** |  |  |  |
| ITA01 |  |  |  | 0.005 | 0.028 | 0.004 |
| ITA02 |  |  |  |  | 0.027 | -0.013 |
| ITA03 |  |  |  |  |  | 0.033 |
