# Supplementary material for: Genetic diversity and differentiation among insular honey bee populations in the southwest Indian Ocean likely reflect old geographical isolation and modern introductions
Source: PLoS One. 2017 Dec 27;12(12):e0189234. doi: 10.1371/journal.pone.0189234 (PMC5744932; doi:10.1371/journal.pone.0189234)
Supplement: S4 Table — Statistical significance for the permutation tests after Bonferroni corrections is indicated in bold (P < 0.000476). Colors as in Table 3. (DOCX) [file pone.0189234.s015.docx]

**S4 Table**

|  | MUS04 | MUS06 | MUS07 | MUS08 | MUS09 | MUS12 | MUS15 | MUS16 | MUS17 | MUS18 | MUS19 | MUS20 | MUS21 | MUS26 |  |  | **0** | |  |
| --- | --- | --- | --- | --- | --- | --- | --- | --- | --- | --- | --- | --- | --- | --- | --- | --- | --- | --- | --- |
| MUS02 | 0.015 | 0.032 | 0.008 | 0.029 | **0.065** | 0.012 | 0.056 | 0.009 | 0.039 | 0.004 | **0.088** | **0.144** | **0.200** | 0.049 |  |  |  | |  |
| MUS04 |  | 0.035 | 0.016 | 0.019 | **0.062** | 0.037 | 0.053 | 0.03 | 0.045 | 0.02 | **0.083** | **0.138** | **0.180** | 0.022 |  |  | **0.1** | |  |
| MUS06 |  |  | 0.046 | 0.042 | 0.075 | 0.054 | **0.051** | 0.022 | 0.036 | 0.014 | 0.089 | **0.156** | **0.194** | 0.036 |  |  |  | |  |
| MUS07 |  |  |  | 0.003 | 0.045 | 0.006 | 0.023 | 0.008 | 0.04 | 0.012 | **0.069** | **0.118** | **0.154** | 0.002 |  |  | **0.2** | |  |
| MUS08 |  |  |  |  | 0.019 | 0.027 | 0.025 | 0.014 | 0.017 | 0.025 | 0.031 | **0.082** | **0.106** | -0.022 |  |  |  | |  |
| MUS09 |  |  |  |  |  | **0.070** | 0.057 | 0.049 | 0.025 | 0.047 | 0.003 | **0.024** | 0.039 | 0.007 |  |  | **0.3** | |  |
| MUS12 |  |  |  |  |  |  | 0.027 | 0.002 | **0.046** | 0.02 | **0.088** | **0.149** | **0.201** | 0.046 |  |  |  | |  |
| MUS15 |  |  |  |  |  |  |  | 0.022 | 0.046 | 0.033 | 0.062 | **0.130** | **0.166** | 0.028 |  |  | **0.4** | |  |
| MUS16 |  |  |  |  |  |  |  |  | 0.021 | 0.006 | **0.065** | **0.129** | **0.173** | 0.027 |  |  |  | |  |
| MUS17 |  |  |  |  |  |  |  |  |  | 0.019 | 0.037 | **0.090** | **0.117** | 0.028 |  |  | **0.5** | |  |
| MUS18 |  |  |  |  |  |  |  |  |  |  | **0.062** | **0.126** | **0.169** | 0.014 |  |  |  | |  |
| MUS19 |  |  |  |  |  |  |  |  |  |  |  | 0.022 | 0.030 | 0.015 |  |  | **0.6** | |  |
| MUS20 |  |  |  |  |  |  |  |  |  |  |  |  | 0.001 | 0.078 |  | | |  |  |
| MUS21 |  |  |  |  |  |  |  |  |  |  |  |  |  | 0.088 |  | | |  |  |
